# Structural Basis of Nucleic Acid Recognition and 6mA Demethylation by *Caenorhabditis elegans* NMAD-1A

**DOI:** 10.3390/ijms25020686

**Published:** 2024-01-05

**Authors:** Guohui Shang, Meiting Yang, Min Li, Lulu Ma, Yunlong Liu, Jun Ma, Yiyun Chen, Xue Wang, Shilong Fan, Mengjia Xie, Wei Wu, Shaodong Dai, Zhongzhou Chen

**Affiliations:** 1 State Key Laboratory of Animal Biotech Breeding and Beijing Advanced Innovation Center for Food Nutrition and Human Health, College of Biological Sciences, China Agricultural University, Beijing 100193, China; 2 National Protein Science Facility, Tsinghua University, Beijing 100084, China; 3 School of Life Sciences, Tiangong University, Tianjin 300387, China; 4 Department of Biochemistry, University of Colorado, Boulder, CO 80303, USA; 5 Department of Pharmaceutical Sciences, Skaggs School of Pharmacy and Pharmaceutical Sciences, University of Colorado Anschutz Medical Campus, Aurora, CO 80045, USA

**Keywords:** NMAD-1A, 6mA demethylation, AlkB homolog, crystal structure, Flips, CTD, NTE, nucleosomes

## Abstract

*N*^6^-methyladenine (6mA) of DNA is an emerging epigenetic mark in the genomes of *Chlamydomonas*, *Caenorhabditis elegans*, and mammals recently. Levels of 6mA undergo drastic fluctuation and thus affect fertility during meiosis and early embryogenesis. Here, we showed three complex structures of 6mA demethylase *C. elegans* NMAD-1A, a canonical isoform of NMAD-1 (F09F7.7). Biochemical results revealed that NMAD-1A prefers 6mA Bubble or Bulge DNAs. Structural studies of NMAD-1A revealed an unexpected “stretch-out” conformation of its Flip2 region, a conserved element that is usually bent over the catalytic center to facilitate substrate base flipping in other DNA demethylases. Moreover, the wide channel between the Flip1 and Flip2 of the NMAD-1A explained the observed preference of NMAD-1A for unpairing substrates, of which the flipped 6mA was primed for catalysis. Structural analysis and mutagenesis studies confirmed that key elements such as carboxy-terminal domain (CTD) and hypothetical zinc finger domain (ZFD) critically contributed to structural integrity, catalytic activity, and nucleosome binding. Collectively, our biochemical and structural studies suggest that NMAD-1A prefers to regulate 6mA in the unpairing regions and is thus possibly associated with dynamic chromosome regulation and meiosis regulation.

## 1. Introduction

*N*^6^-methylation of nucleic acid bases increases the abundance of genetic information carried in DNA or RNA sequences. DNA *N*^6^-methyladenine (6mA) modification is the most prevalent DNA modification in prokaryotes [1,2,3] but is rare in eukaryotes [4,5,6]. Many publications have identified 6mA DNA modifications in different eukaryotic species [7,8,9,10,11,12,13,14,15,16]. Several reports have already identified 6mA as a novel DNA modification in metazoans [8,9]. Recent studies have shown that DNA 6mA is involved in gene regulation, transposons, stem cell differentiation, and human tumors and plays critical roles in cell biology [12,13,17,18]. Obviously, 6mA is an emerging epigenetic mark in the eukaryotic genome and plays a significant role in many fields.

The AlkB family of dealkylating enzymes, α-ketoglutarate (α-KG) and Fe(II)-dependent dioxygenases, are evolutionarily conserved. They can demethylate a diverse repertoire of methylated DNA, including 1mA, 6mA, and RNA m^6^A in a variety of species [19,20,21]. The AlkB family members have broad substrate selectivities based on flexible loops and non-homologous domains [22]. There are nine AlkB homologs including ALKBH1–8 and fat mass and obesity-associated (FTO) with low sequence identity [23,24] in humans. These nine human AlkB homologs have abundant functions in DNA repair, RNA stability, nuclear transport, cancers, viral infection, and fatty acid metabolism [25,26,27]. In addition, we previously determined structures of human ALKBH1, and ALKBH5–7 [28,29,30,31], providing precise structure information for understanding protein function.

In *Caenorhabditis elegans*, the global level of DNA 6mA increases in response to mitochondrial stress, reflecting the significance of the 6mA modification in stress response [7,32]. NMAD-1, one of the AlkB family members in *C. elegans*, demethylates the DNA damage modification such as 6mA and 3mC [12,33]. A few studies also demonstrated that NMAD-1 was indispensable for DNA replication during meiosis in the germline [33]. However, whether more suitable biochemical substrates of NMAD-1 exist and the underlying molecular basis remain obscure so far.

Here, we show three *C. elegans* canonical isoform NMAD-1A crystal structures with different ligands, including Mn^2+^/α-KG, Mn^2+^, or SO_4_^2−^. These structures present a conventional and conserved double-stranded β-helix (DSBH) consistent with multiple unique structural features of a stretch-out Flip2 motif and a functionally indispensable carboxy-terminal domain (CTD). By establishing a reliable assay in vitro, we provide key evidence on novel features of NMAD-1A substrates by analyzing structural features, characterized by a locally unpairing structure that contains a flipped 6mA base such as Bubble/Bulge DNAs [30,34,35,36,37,38,39,40] instead of 6mA ssDNA or dsDNA, revealing its role in DNA mismatch repair [41]. Notably, NMAD-1A has relatively higher activity on 6mA Bulge6 DNA, which is more than three times that on ssDNA.

## 2. Results

### 2.1. NMAD-1A Prefers Bubble/Bulge DNAs as Substrates

**Figure 1:**
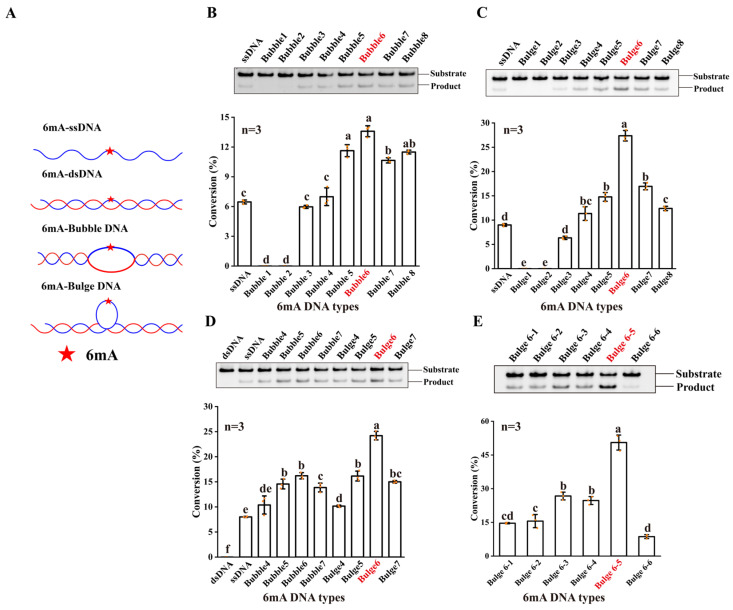
The DNA 6mA demethylation activity of NMAD-1A. (**A**) ssDNA and DNAs with different secondary structures. Substrates are shown as cartoons and 6mA is shown as a red star. Detection of 6mA demethylation assays (**top**) of ssDNA, Bubble DNAs (**B**), Bulge DNAs (**C**), and corresponding statistical analysis (**bottom**). Detection of 6mA demethylation assays of dsDNA, ssDNA, Bubble 4–Bubble 7 DNAs, and Bulge 4–Bulge 7 DNAs (**D**); Bulge 6-1–Bulge 6-6 DNAs (**E**). All reactions for 4 h by WT NMAD-1A using *Dpn* II. ((**B**–**E**), **bottom**), n = 3 biologically independent experiments (shown as orange dots). Substrates with higher activity are marked in red. Conversion (%), the proportion of product. Data are presented as mean ± SD, and different letters (a, b, c, d, e, f) indicate significant differences among groups (one-way analysis of variance (ANOVA)), *p* < 0.05.

Given that the HPLC method was not sensitive and quick enough to detect the demethylation activity of NMAD-1A, we employed a high-throughput methylation-sensitive restriction digest assay toward 6mA ss/ds/Bubble/Bulge DNAs (Figure 1A and Figures S1 and S2) using nuclease *Dpn* II [25,42]. Enzymatic profiling studies showed that the demethylation activity of wild-type (WT) NMAD-1A on ssDNA was low (Figure 1B,C), probably because ssDNA was not the most suitable substrate. Moreover, NMAD-1A had no demethylation activity on 6mA dsDNA (Figure 1D).

We then introduced various substrates, such as hemi-methylated Bubble and Bulge DNAs with different numbers of mismatched base pairs in the middle of double-stranded DNAs (Figure 1A). Intriguingly, NMAD-1A displayed higher demethylation activities for Bubble/Bulge DNAs (Figure 1C,D). The demethylation activity of NMAD-1A on Bubble 6 DNA was twice that on ssDNA when the number of mismatched base pairs ranged from 5 to 7 (Figure 1D). Notably, NMAD-1A had the strongest activity on Bulge 6 DNA, which was threefold that toward ssDNA (Figure 1D). Furthermore, we tested the demethylation activity toward Bulge DNAs with 6mA at different mismatch positions and found that the activity was the highest at the fifth mismatched base pair, Bulge 6-5 DNA (Figure 1E). That is to say, Bulge 6-5 DNA might be more suitable to penetrate the active center.

### 2.2. Rational Design of NMAD-1A Mutations Facilitates the Crystallization of NMAD-1A

**Figure 2:**
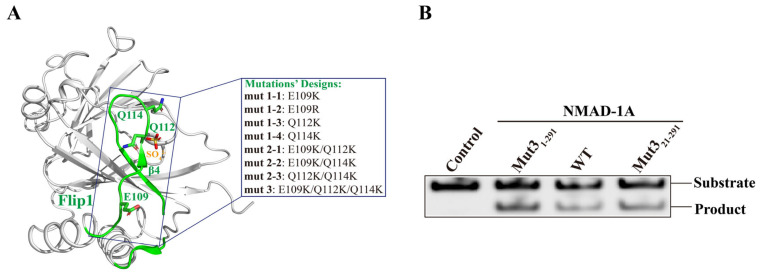
Rational design of NMAD-1A mutations used for crystallization. (**A**) The related mutations of the Flip1 region from the construct NMAD-1A_21-263_ structure. (**B**) In vitro demethylation assays of NMAD-1A and the mut3-NMAD-1A_1-291_/NMAD-1A_21-291_ reaction for 45 min toward substrate 6mA Bulge 6-5 DNA. Three biological replicates are performed.

At first, we obtained a structure of the WT construct NMAD-1A (residues 21–263) missing the CTD at the resolution of 2.7 Å after extensive screening of protein constructs and crystallization trials (Figure 2A). Surprisingly, the active center in this structure was distorted as reflected by the flipping of Asp-186 away from the active site (Figure S3A), indicating the importance of the CTD. To obtain a structure with a typical active site’s conformation, we used full-length NMAD-1A or certain NMAD-1A constructs containing the CTD for additional crystal screening. However, all experiments to obtain crystals suitable for X-ray diffraction failed. Finally, we chose to engineer NMAD-1A with site-directed mutagenesis to increase the binding ability of NMAD-1A to nucleic acids, thus making protein crystallization easier [43].

Based on the structure of WT NMAD-1A_21-263_-SO_4_^2−^, we generated NMAD-1A variants with site-directed mutations, which were selected according to the following criteria: (i) increased binding affinity to short nucleotides and (ii) without lower demethylation activity. We then expressed and purified these NMAD-1A mutants involving three mutations (E109K, Q112K, Q114K) separately or jointly from the Flip1 region (Figure 2A) and determined their binding ability to different short nucleotides (Table S1) using FPLC. Next, we collected the peaks of the protein and nucleotide complex for crystallization. Finally, the mutants E109K/Q112K/Q114K (mut3) NMAD-1A_1-291_/NMAD-1A_21-291_ maintained the enzymatic demethylation activity (Figure 2B) and were observed to significantly increase the binding affinity to nucleotides for protein crystallization compared with WT NMAD-1A (Figures S3 and S4D–I).

### 2.3. The Overall Structure of NMAD-1A

**Figure 3:**
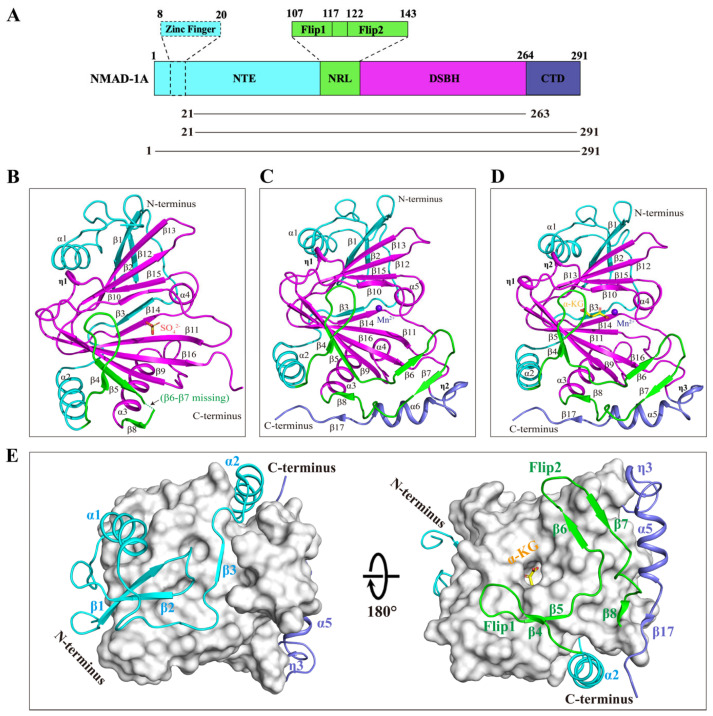
The overall structures of NMAD-1A with SO_4_^2−^, Mn^2+^, or Mn^2+^-α-KG. (**A**) Schematic domain architecture of NMAD-1A (**top**) and constructs used in this study (**bottom**). The NTE (residues 1–106), NRL (residues 107–143), DSBH (residues 144–263), and CTD (residues 264–291) are colored in cyan, green, magenta, and slate, respectively. The Flip1 and Flip2 regions of the NRL domain are shown in a zoom-in box. The hypothetical ZFD (residues 8–20), dotted box. Cartoon representations of the structures of NMAD-1A_21-263_-SO_4_^2−^ (**B**), the mut3-NMAD-1A_21-291_-Mn^2+^ (**C**), and mut3-NMAD-1A_1-291_-Mn^2+^-α-KG (**D**). SO_4_^2−^, orange; β6-β7 missing, green dashed line. Mn^2+^, purple-blue; α-KG, yellow. The spatial connection of DSBH with NTE, NRL, and CTD (**E**). DSBH is shown as a light gray surface. NTE, cyan, NRL, green, CTD, slate.

*C. elegans* NMAD-1A protein has 291 residues involving an N-terminal extension (NTE), a nucleotide-recognition lid (NRL), a DSBH domain, and the CTD. The NTE contains a hypothetical ZFD, and the NRL contains Flip1 and Flip2 regions (Figure 3A).

To elucidate the molecular mechanisms underlying NMAD-1A function, we finally determined three crystal structures, 2.7 Å NMAD-1A_21-263_-SO_4_^2−^, 3.1 Å mut3 NMAD-1A_21-291_-Mn^2+^, and 3.0 Å mut3 NMAD-1A_1-291_-Mn^2+^-α-KG (Figure 3B–D and Figure S5 and Table S2). Although we finally obtained the structure of mut3 NMAD-1A_1-291_ full-length crystals (Figure S4H,I), the ZFD exhibited poorly defined electron density. Then, the refined overall structure was similar to the mut3 NMAD-1A_21-291_ structure without the ZFD owing to its flexibility (Figure 3C,D). In addition, SAXS analysis [44,45] showed that NMAD-1A existed mainly in the form of monomer in solution (Figure S6 and Table S3). Hence, four copies of NMAD-1A found in each asymmetric unit were due to the crystal packing (Figure S7).

Here, we illustrate the structural features of NMAD-1A by the mut3 NMAD-1A_1-291_-Mn^2+^-α-KG structure mainly. The DSBH, as the catalytic core of NMAD-1A, adopts a typical eight-stranded (β9–β16) jelly-roll fold that contains a major sheet of antiparallel β-strands (β9–β16–β11–β14) and a minor sheet (β10–β15–β12–β13) to sandwich Mn^2+^ and α-KG ligands at the active center, a large α-helix α3, a small α-helix α4, and two 3_10_ helixes (η1–η2) (Figure 3D). Additionally, the DSBH is notably wrapped around and stabilized by additional elements from the NTE, NRL, and CTD, with the NTE domain packing on one side and the NRL domain and the CTD on the opposite side (Figure 3E). In general, the DSBH of NMAD-1A is conserved in the Fe(II)/α-KG-dependent dioxygenase superfamily. DALI search (http://www2.ebi.ac.uk/dali accessed on 27 December 2021) reveals that ALKBH5 (PDB: 4NPL) is the most similar structural homolog of the NMAD-1A in the PDB database, with a Z-score of 18.6 and a root mean squared deviation (r.m.s.d.) of 2.5 Å over 173 Cα atoms. There are also structural similarities of NMAD-1A with other determined human AlkB family members, such as ALKBH8 (PDB: 3THT) (Z-score 16.5 and r.m.s.d. 2.8 Å), ALKBH3 (PDB: 2IUW) (Z-score 16.1 and r.m.s.d. 2.8 Å), and ALKBH7 (PDB: 4QKB) (Z-score 15.9 and r.m.s.d. 2.6 Å).

### 2.4. SO_4_^2−^ Is a Potential Ligand Affecting NMAD-1A’s Conformation

**Figure 4:**
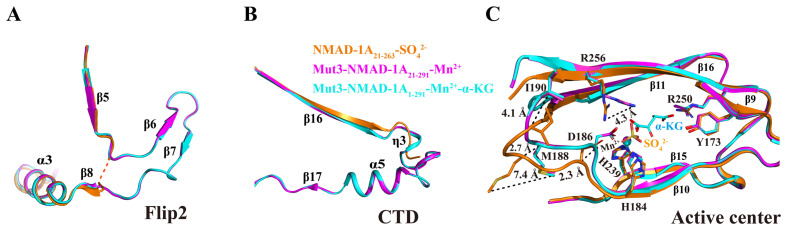
The key role of CTD in stabilizing the structure of NMAD-1A. The Flip2 region (**A**), CTD (**B**), and active center (**C**) differences between these above three structures.

Here, we compared the three structures determined above. For the NRL domain, the Flip2 region was incomplete with missing β6–β7 strands and adjacent loops in the structure of NMAD-1A_21-263_, while it was intact in both the mut3 NMAD-1A_21-291_-Mn^2+^ and mut3 NMAD-1A_1-291_-Mn^2+^-α-KG (Figure 4A). Furthermore, NMAD-1A_21-263_ also lacks the CTD, including three secondary structure elements: a 3_10_ helix (η3), α5, and β17 (Figure 4B). In the active center, the side chain NH_2_ of Arg-256 in the structure of NMAD-1A_21-263_ flips away for 4.3 Å compared with that of the mut3-NMAD-1A_1-291_/NMAD-1A_21-291_. In addition, the main chains of Asp-186, Met-188, and Ile-190 in the structures containing the CTD further moved into the active center 2.3 Å, 2.7 Å, and 4.1 Å, respectively (Figure 4C). Moreover, the spatial positions of the three mutational residues (Glu-109, Gln-112, and Gln-114) in mut3-NMAD-1A_1-291_/NMAD-1A_21-291_ structures were not changed and far away from the CTD, revealing that these structural changes did not arise by the designed mutations.

All the above analyses indicate that SO_4_^2−^ may induce many conformational changes of the active center (Figure 4C). In support, the structure of NMAD-1A_21-263_ demonstrated that the SO_4_^2−^ ligand resulted in a significant distortion of the cofactor coordination geometry as reflected by flipping away Asp-186 and the loop containing the conserved Hx(D/E) motif of the active center (Figure 4C). Neither α-KG nor Mn^2+^ but SO_4_^2−^ ligand was observed in the NMAD-1A_21-263_ catalytic center, whose His-239 was different from the corresponding His-287 around the N-truncated ALKBH1_37-369_ (Δα1) and nearly identical to ALKBH1_1-359_ [40] (Figure S8). This result revealed that SO_4_^2−^ might act as a potential ligand, leading to the conformation distortion of the construct NMAD-1A_21-263_.

### 2.5. NMAD-1A Binds to α-KG and Mn^2+^ in the Conserved Active Center

**Figure 5:**
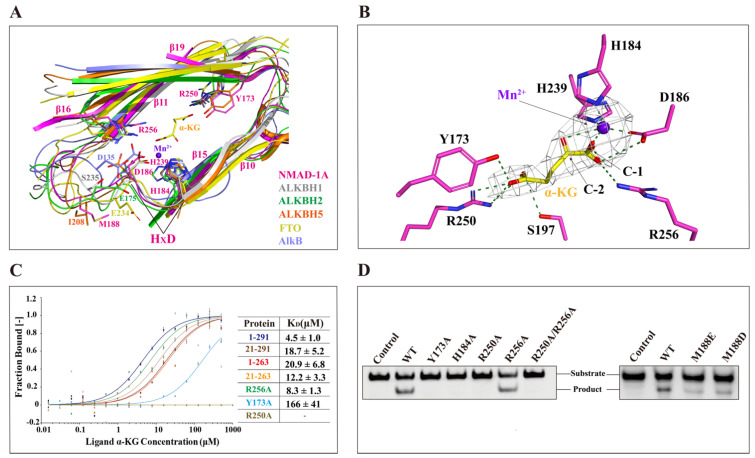
The unique active center of NMAD-1A. (**A**) The unique active sites of NMAD-1A resemble AlkB (PDB: 3BIE, slate), ALKBH1 (PDB: 6IE2, gray), ALKBH2 (PDB: 3BTY, green), ALKBH5 (PDB: 4NRM, orange), and FTO (PDB: 3LFM, yellow). The conserved HxD motif sequence is indicated and marked with black lines, and the key residues are displayed with sticks. The α-KG and Mn^2+^ in the NMAD-1A structure are represented by yellow sticks and purple-blue spheres, respectively. (**B**) Detailed interactions of NMAD-1A with α-KG and Mn^2+^. Hydrogen bonds, green dashes; the *Fo-Fc* omit map of α-KG and Mn^2+^ (2.5 σ). (**C**) Measurement of the binding affinities of NMAD-1A and mutants to α-KG by MST. (**D**) Mutations of the key residues in the active center or near the active sites greatly impair the NMAD-1A demethylation assays for 2 h toward substrate 6mA Bulge 6-5 DNA. Three biological replicates are performed.

Like other AlkB family proteins, NMAD-1A requires the binding of the cosubstrate α-KG and ferrous iron for catalysis (Figure 5A) [12]. In the NMAD-1A_1-291_-Mn^2+^-α-KG complex structure, the Mn^2+^ ion is in an octahedral geometry and coordinated by NE2 atoms of His-184 and His-239, carboxylate oxygen of Asp-186, and the C1-carboxylate and C2-carbonyl groups of α-KG, respectively (Figure 5B). Based on the sequence alignment of the other AlkB family members, His-184, Asp-186, and His-239 (so-called HxD…H motif) of NMAD-1A are conserved across the AlkB family (Figure S9). Therefore, NMAD-1A, like other AlkB family members, binds to the metal ion in a conserved manner in the DSBH domain. The binding of α-KG is also apparently stabilized by hydrogen bonds with the side chains of Tyr-173, Asp-186, Ser-197, and His-239 and salt bridges involving the side chains of Arg-250 and Arg-256 in the NMAD-1A active center (Figure 5B). Moreover, both Arg-250 and Arg-256 are also well conserved in the AlkB family (Figure S9), revealing their conservation and importance.

Interestingly, sequence alignment reveals that all these above key residues are highly conserved among NMAD-1 orthologs in different species (Figure S10) as well as in other Fe(II)/α-KG-dependent dioxygenases (Figure S9) [22,46]. By designing several alanine substitutions of these key residues involved in binding the metal ion Fe^2+^ and α-KG, we found that the related mutations except R256A abolished the demethylation activity on 6mA DNA (Figure 5C,D), further indicating the important roles of the conserved active center.

Active center alignment of NMAD-1A with ALKBH1, ALKBH2, ALKBH5, FTO, and AlkB revealed potential residues involved in 6mA interaction (Figure 5A). In particular, the residue Met-188 of NMAD-1A overlapped with the Ile-208 of ALKBH5 [47] (Figure 5A). Since the ALKBH5 mutants I208D and I208E showed decreased 6mA demethylation activity [47], Met-188 of NMAD-1A might also play a similar role in sandwiching 6mA for demethylation. Additionally, the Met-188 of NMAD-1A took a significant conformational change in the presence of SO_4_^2−^ and was very conserved among sequence alignments of NMAD-1A orthologs (Figure 4C and Figure S10). Consistently, M188D/M188E mutation resulted in a dramatic activity loss (Figure 5D), likely due to the weaker hydrophobic interaction in binding the neutral 6mA base by changing the neutral side chains to negatively charged ones [48,49,50,51].

### 2.6. The CTD Is a Key Domain for Demethylating Substrates and Binding Nucleosomes

**Figure 6:**
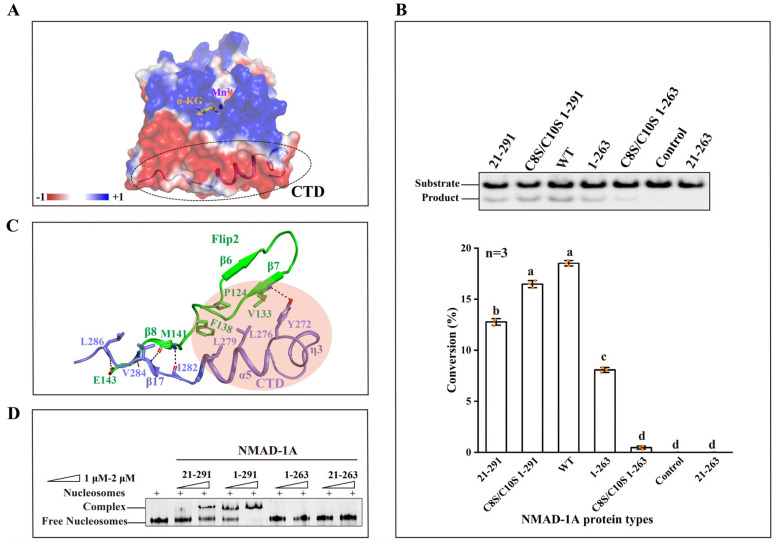
The CTD is vital for demethylation activity and the binding to nucleosomes. (**A**) Electrostatic surface representation of NMAD-1A (red, negative; blue, positive; light gray, neutral). The electrostatic surface of CTD is mostly negative highlighted by a dashed ellipse. (**B**) In vitro demethylation assays of WT NMAD-1A, CTD, and ZFD-related mutants for 1 h toward substrate 6mA Bulge 6-5 DNA (**top**). The corresponding statistical analysis (**bottom**), n = 3 biologically independent experiments (shown as orange dots). Substrates with higher activity are marked in red. Conversion (%), the proportion of product. Data are presented as mean ± SD, and different letters (a, b, c, d) indicate significant differences among groups (one-way analysis of variance (ANOVA)), *p* < 0.05. (**C**) Detailed interactions between CTD and Flip2. Hydrophobic contacts are indicated with a pink circle, hydrogen-bonding interactions are indicated with black dashed lines. (**D**) Electrophoretic mobility shift assay (EMSA) of WT or different truncated NMAD-1A with the nucleosomes.

The CTD of NMAD-1A is far away from the active center (Figure 3C,D and Figure 6A), however, its loss dramatically compromises the demethylation activity (Figure 6B). To know the reason why it affects the catalytic activity, we performed structural analyses. From the NMAD-1A_21-263_-SO_4_^2−^ structure, we found that the Flip2 region of NRL was incomplete, probably due to the instability of this region (Figure 4A and Figure S11C). However, the Flip2 region of NRL from the mut3 NMAD-1A_1-291_ or NMAD-1A_21-291_ structure was intact with well-defined electron density (Figure 3C,D and Figure 4A). As calculated by the PISA server (https://www.ebi.ac.uk/msd-srv/prot_int/pistart.html) (accessed on 1 December 2022), the CTD interacts with NMAD-1A_21-263_ with a large interface area of 959.7 Å^2^ (Table S4). Notably, the CTD interacts with and further stabilizes the Flip2 region by hydrophobic contacts, including Tyr-272, Leu-276, and Leu-279 of the CTD as well as Pro-124, Val-133, and Phe-138 of the Flip2 region (Figure 6C). Most of these residues are highly conserved across NMAD-1A orthologs (Figure S10). In addition, the main chains of Ile-282, Val-284, and Leu-286, together with the side chain of Tyr-272 of the CTD, form several hydrogen bonds with the main chains of Met-141, Glu-143, and Val-133 and the side chain of Glu-143 of the Flip2 (Figure 6C). Collectively, we suggested that the CTD of NMAD-1A is essential for the Flip2 region. Also, the sequence of the CTD is very distinctive based on the structure-based sequence alignment of NMAD-1A with other AlkB family members (Figure S9). Compared with the WT NMAD-1A_21-291_ construct, the CTD-deleted construct WT NMAD-1A_21-263_ was deprived of the activity completely (Figure 6B), suggesting that the CTD was key for the catalytic activity through the formation of a substrate recognition interface with the Flip2 region. Here, we found that NMAD-1A bound nucleosomes in vitro for the first time (Figure 6D), consistent with its regulation of chromosomal segregation in meiosis [33]. In addition, due to the continuously negative electrostatic potential surface, the CTD is also vital for the interaction between NMAD-1A and nucleosomes (Figure 6A,D).

### 2.7. The Distinct Structural Features of the NTE

The NTE of NMAD-1A consists of an unsolved ZFD, three β-strands (β1–β3), two α-helixes (α1–α2), and the adjacent loops (Figure 3B–D). These elements wrap around the DSBH and have spatial specificity (Figure 3E).

Enzymatic profiling studies revealed that the ZFD promoted the 6mA demethylation activity of NMAD-1A in vitro, as WT full-length NMAD-1A_1-291_ had higher activity than the ZFD-deleted construct NMAD-1A_21-291_ (Figure 6B). Additionally, for the constructs lacking the CTD, we surprisingly found that the NMAD-1A_1-263_ retaining the ZFD exhibited a decreased 6mA demethylation activity while the NMAD-1A_21-263_ lost it totally (Figure 6B). Moreover, the demethylation activity of ZFD mutants C8S/C10S of WT NMAD-1A_1-291_/NMAD-1A_1-263_ decreased, too (Figure 6B). In addition, the ZFD collaboratively promoted the nucleosome binding, probably owing to its positive electrostatic surface (Figure 6D and Figure S15A). This suggested the importance of the ZFD in activity and dynamic chromosome regulation for NMAD-1A.

**Figure 7:**
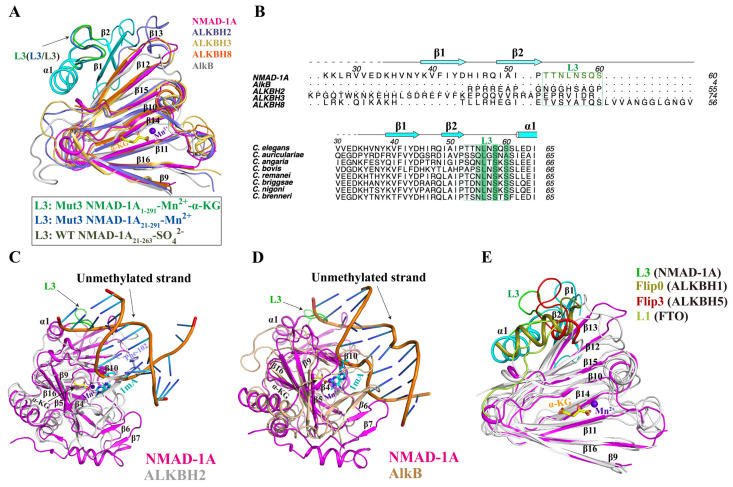
The NTE loop L3 of NMAD-1A is important for its selection against dsDNA. (**A**) Structural comparison of NMAD-1A with *E. coli* AlkB (PDB: 3BIE), human ALKBH2 (PDB: 3S57), ALKBH3 (PDB: 2IUW), and ALKBH8 (PDB: 3THP) around the DSBH. The NTE loop L3 (residues 53–60) from the mut3-NMAD-1A_1-291_, the mut3-NMAD-1A_21-291_, and WT NMAD-1A_21-263_ are shown in green, blue, and gray, respectively. (**B**) Structure-based sequence alignment of NMAD-1A orthologs, *E. coli* AlkB, human ALKBH2, ALKBH3, and ALKBH8 within the NTE. The conserved residues are colored green. Structural alignment of NMAD-1A with complex ALKBH2-dsDNA (PDB: 3BTY) [52] (**C**) and AlkB-dsDNA complex (PDB: 2FD8) [48] (**D**). Phe-102 of ALKBH2, slate sticks. (**E**) Structural comparison of NMAD-1A with human ALKBH1 (PDB: 6IE2), ALKBH5 (PDB: 4NRM), and FTO (PDB: 3LFM) around the DSBH. The extra domains against dsDNA from NMAD-1A, ALKBH1, ALKBH5, and FTO are colored in green, deep olive, red, and lime, respectively.

The loop L3 of NTE has had its special structure and sequence features compared with human ALKBH2, ALKBH3, ALKBH8, and *E. coli* AlkB and is relatively conserved among NMAD-1A orthologs (Figure 7A,B). Moreover, the positions of the L3 are the same between the determined three NMAD-1A structures and are probably fixed by forming interactions with the DSBH domain. Moreover, low temperature factors of the L3, β1, and α1 reveal their stability in the structure (Figure 7A and Figure S11C). To understand why NMAD-1A did not demethylate paired dsDNA (Figure 1D), we compared the structure of NMAD-1A with those of ALKBH2-dsDNA [48] and AlkB-dsDNA [52] and found that the loop L3 impeded the access of paired dsDNA to the active site (Figure 7C,D). A similar phenomenon was also observed in ALKBH5 [28,53], FTO [54], and ALKBH1 [30] (Figure 7E). Thus, the loop L3 is probably key for its selection against dsDNA. In addition, α1 and two β-strand (β1 and β2) elements of the NTE show their distinct features compared with the other AlkB members (Figure S12C,D). In addition, two NTE-deletion mutant constructs Δα1 (62–74) and Δβ1–β2 (21–52) could not obtain soluble proteins using a prokaryotic expression system (Figure S13), revealing their importance in the folding. All the above results suggested the significant role of the spatial connection of DSBH with NTE in stabilizing the integrity and function of NMAD-1A.

### 2.8. The Variable NRL Forms a Unique Substrate-Binding Channel of NMAD-1A

The AlkB family proteins bind and immobilize substrates through NRLs, containing several key loops around the catalytic domain [22]. The NRLs contribute to substrate selectivity [55] and are less conserved among AlkB members. To characterize the differences between NMAD-1A and the other AlkB proteins, we overlaid their structures (Figure S12). The Flip1 and Flip2 of NMAD-1A (Figure 3C,D) form the NRL domain, and their structures are notably different from those of other human AlkB family proteins (Figure S12A,B).

The Flip1 region consists of a very short β-strand β4 and the adjacent loops, exposed to the solvent used for the substrate selectivity (Figure 3B–D). Only one basic residue (Arg-117) is found in the Flip1 region (Figure S9), which may suggest weak binding to nucleic acids. The Flip2 region of NMAD-1A is mainly composed of three β-strands including β6, β7, and a very short β8 from the mut3-NMAD-1A_21-291_/NMAD-1A_1-291_ structures but not the WT NMAD-1A_21-263_ (Figure 3B–D). The Flip2 region extrudes from the surface to form a positive “horn” (Figure 3C,D and Figures S11A and S12B), which may also contribute to the substrate selectivity and binding. Notably, the strand β5 links the Flip1 and the Flip2 region, packs antiparallelly to strand β9, and extends the major β-sheet of the DSBH.

**Figure 8:**
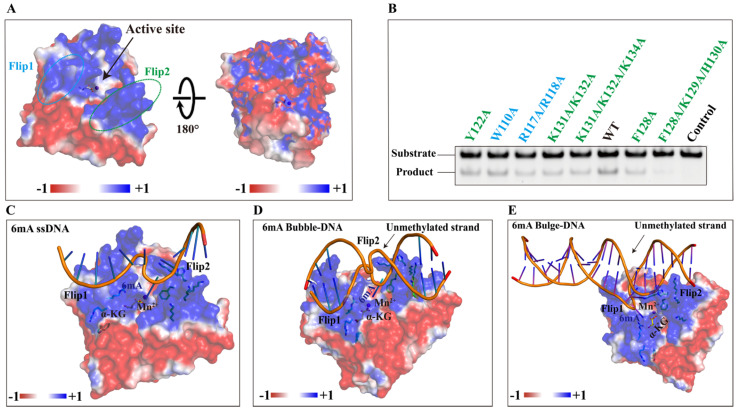
The variable NRL of NMAD-1A decides its unique binding model. (**A**) Potential surface of NMAD-1A with the Flip1 and Flip2 motifs highlighted by dashed ellipses. (**B**) In vitro demethylation assays of NMAD-1A and mutants. Residues of the Flip1, light blue; Flip2, green. Demethylation assays for 2 h toward substrate 6mA Bulge 6-5 DNA. Three biological replicates are performed. The binding models of NMAD-1A and 6mA-modified ssDNA (**C**), 6mA-modified Bubble DNA (**D**), and 6mA-modified Bulge DNA (**E**). α-KG (yellow), Mn^2+^ (purple), Flip1, and Flip2 are shown.

We failed to obtain the NMAD-1A-6mA-containing DNA complex structure after all attempts, which prevented us from making an in-depth analysis of methyl base recognition in a native state. We then turned to structure-based comparison and mutagenesis studies in Flip1 and Flip2 to explore the methyl base-binding pocket of NMAD-1A. R117A/R118A double mutation in Flip1 reduced the demethylation activity. K131A/K132A or F128A mutations in Flip2 also compromised the demethylation activity and, especially, F128A/K129A/H130A triple mutation almost completely abolished the demethylation activity (Figure 8A,B). Thus, based on these data, we believed that Flip1 and Flip2 were involved in substrate interactions. Models of ssDNA/Bubble DNA/Bulge DNA binding to NMAD-1A are shown in Figure 8C–E, respectively.

## 3. Discussion

As reported, human AlkB homologs can exert demethylation activity on ssDNA, dsDNA with a modified base such as 1mA or 6mA, or m^6^A RNA [56,57]. For example, ALKBH1 prefers Bubble or Bulge DNA [30,39,40]; ALKBH2 binds 1mA dsDNA [58]; ALKBH3 chooses ssDNA or RNA as substrate [59,60,61]; ALKBH5, ALKBH8, and FTO function as RNA demethylases [24,28,53,54,62]. Recently, several studies reported that the demethylation activity of NMAD-1A on 6mA DNA was low [12,33,63]. One possible reason could be that the substrates mentioned above were not native and suitable. However, the more suitable substrate of NMAD-1A has been a mystery until now. Here, we developed methylation-sensitive restriction digest assays to evaluate the 6mA demethylation activity of NMAD-1A and succeeded in increasing the binding affinity and activity. Moreover, we reported three structures and suitable substrates of NMAD-1A such as Bubble/Bulge DNAs and nucleosomes. NMAD-1A was found to bind nucleosomes with high affinity for the first time (Figure 6D), consistent with its critical physiological functions in DNA replication and chromosomal segregation during meiosis [33].

In general, a target-modified base can insert the substrate-binding pocket formed by the DSBH and NRL domains of AlkB family members, with its alkyl group embedded inside the active center. Similar to other AlkB homolog dioxygenases, the DSBH domain of NMAD-1A acts as the catalytic core and binds to α-KG ligand and the metal ion in a conserved manner with key and conserved Hx(D/E) motif linking the canonical second (II) and third (III) strands of the DSBH domain (Figure 5A,B,D and Figure 7A and Figures S9 and S10) [64,65]. Interestingly, the CTD-deleted NMAD-1A_1-263_ and NMAD-1A_21-263_ constructs still retained the binding affinity to α-KG ligand, indicating that the DSBH domain might maintain the structural integrity in the absence of the CTD (Figure 5C), consistent with the recently reported conclusion in binding NOG [63].

In the loop containing the conserved Hx(D/E) motif of most AlkB family members, there is at least one polar residue (Asp-135 in AlkB, Glu-175 in ALKBH2, Ser-235 in ALKBH1, and Glu-234 in FTO) that forms an important hydrogen bond with the nucleobase in selecting differently methylated nucleobases [40,54,58,59,66] (Figure 5A). But in the same spatial region, Met-188 of NMAD-1A is hydrophobic, overlapping well with the Ile-208 of ALKBH5 [47] (Figure 5A). Moreover, the M188D/M188E mutation caused a dramatic decline in activity (Figure 5D). This might be structurally explained by the fact that the residues adjacent to the key motif Hx(D/E) in the substrate catalysis center of NMAD-1A are rather hydrophobic (Figure 4A).

The NRL domain of NMAD-1A also shows several unique features (Figure S12A,B). Firstly, the Flip1 region of NMAD-1A is a shorter one, leaving a large vacancy for substrate binding among the family members (Figure 3B–D and Figure S12A), and contains very few basic and aromatic residues (Figure S9) corresponding to Phe-102 in ALKBH2 [58,67] intercalating into the duplex stack and covering the DNA gap (Figure 7C). These features might rationalize the weak demethylation activity of NMAD-1A [68]. Secondly, the distance between the Flip2 region and the opposing edge of the cleft is only 7.7 Å (measured between Phe-128 Cα and Trp-189 Cα) (Figure S11B). This is suitable for ssDNA or ssRNA to fit between the rims of the putative binding cleft. Noteworthily, there are relatively high B-values for residues in the Flip2 region and the width of the channel between the Flip1 and Flip2 of NMAD-1A (~18.7 Å) has enough space to accommodate the Bubble/Bulge/ssDNA (Figure S11A,C), as observed in the corresponding flexible loop (the Flip2 region) in the AlkB complex (Figure S11D) [58]. In summary, our research revealed that the more suitable substrates of NMAD-1A were Bubble/Bulge DNAs theoretically anchored by the short Flip1 and the horned Flip2 (Figure 1 and Figures S1, S2 and S12A,B).

Additionally, the loop L3 of NMAD-1A, similar to the Flip0 (ALKBH1) [30], loop L1 (FTO) [54], and Flip3 (ALKBH5) [54], is probably necessary for its selection against paired dsDNA by structural alignments (Figure 7C–E). Moreover, the NMAD-1A binds paired dsDNA but without activity (Figure 1D), revealing a non-productive mode, rather than a productive mode [69]. This might shed light on the design of new agonists and antagonists for the AlkB family [70].

The hypothetical ZFD of NMAD-1A is unique among the AlkB family (Figure S9). Based on the electron density map of mut3-NMAD-1A_1-291_-Mn^2+^-α-KG containing a very disorderly and discontinuous ZFD compared with the structure of the mut3-NMAD-1A_21-291_-Mn^2+^, the structure of the ZFD was unstable without the substrate. The absence of the ZFD in our mut3-NMAD-1A_1-291_ structure might be due to its flexibility (Figure S14), a similar observation to the recently determined truncated structure NMAD-1A_32-291_ (PDB: 8H68) removing residues (1–31) to facilitate the crystallization [63]. To detect the conformation of the ZFD, we performed SAXS analysis of WT full-length NMAD-1A_1-291_ in solution. WT NMAD-1A_1-291_ existed mainly in the form of 93.3% monomers and 6.7% dimers, corresponding to a wide peak of 4 mL in the size exclusion chromatography (Figures S6A and S15B and Table S3). Furthermore, the low-resolution ZFD solution structure of WT NMAD-1A_1-291_ was shown using SAXS methods [71,72] (Figure S15C–E). Furthermore, full-length NMAD-1A_1-291_ had higher demethylation activity and nucleosome-binding ability compared with the ZFD-lacking construct NMAD-1A_21-291_ (Figure 6B–D), suggesting that the ZFD indeed contributed to binding nucleic acids and was distinctive from the C-terminal Zn (II)-binding site of ALKBH8 for structure stabilization [62]. This function was reported in the AlkB family for the first time. Thus, the unsolved ZFD structure requires further investigation in the future.

The CTD is vital for stabilizing the Flip2 region by conserved and extensive interactions from the mut3-NMAD-1A_1-291_/NMAD-1A_21-291_ structures (Figure 6C and Figure S10 and Table S4). The CTD-deleted construct NMAD-1A_1-263_ decreased the demethylation activity sharply (Figure 6B). This suggests that the CTD function of NMAD-1A corresponds to FTO whose CTD forming a three-helix bundle plays an important role in interacting and stabilizing the conformation of the NTD essential for FTO demethylation activity [24,43,54]. In summary, the CTD is vital for maintaining the demethylation activity of NMAD-1A.

Taken together, our results show that both the NTE and CTD are important for stabilizing the overall structure (Figure 4A and Figure 6C and Figure S13). Furthermore, the NTE and CTD are also vital for the demethylation activity (Figure 6B). Moreover, residues in the NTE and CTD are mostly conserved among sequence alignments of NMAD-1A orthologs (Figure S10), suggesting that the structures and functions of the NTE and CTD are conserved in NMAD-1A from different species. Thus, our findings can be used to further study the regulatory mechanisms of 6mA modification in different basic biological processes and in the field of DNA epigenetics to guide future drug research.

## 4. Notes on the Structure of Maltose-Binding Protein (MBP)-Fused NMAD-1A_32-291_

When we were preparing our manuscript, another group released the structure of NMAD-1A_32-291_ (PDB: 8H68) [63]. A quick comparison with our structures indicates that the whole structures are similar. However, the sequence lengths of proteins in our structures are longer. NMAD-1A was reported to be unable to bind various DNA oligos, including ssDNAs, dsDNAs, and dsDNAs with or without a single mismatch at the 6mA site in EMSA and MST measurements [63]. Here, we succeeded in increasing the binding affinity after optimizing a series of conditions such as buffer types, pH, temperatures, and substrates such as nucleosomes.

Furthermore, our results reveal that the CTD is critical in binding nucleosomes and demethylating substrates. Moreover, our structures also revealed a large active center’s conformational change required for the DNA 6mA demethylation including substrate identification and binding. Thus, the major conclusions in our manuscript are not shown in the structure PDB code 8H68. We believe our structures are more physiologically relevant than the aforementioned NMAD-1A structure and closest to human ALKBH4 which might function in the demethylation of 6mA DNA or methylated actin [73,74].

## 5. Materials and Methods

### 5.1. Protein Expression and Purification

DNAs encoding wild-type (WT) and mutants of *C. elegans* NMAD-1A were amplified by PCR and subcloned into the pET-28a (Novagen) vector containing a protease recognition site of tobacco etch virus (TEV) [75] fused with a 6×His Tag at the N-terminus. The final clones were verified by DNA sequencing. All recombinant plasmids were transformed into *E. coli* BL21 (DE3). The cells were grown in LB at 37 °C until the absorbance at 600 nm (A_600_) reached 0.8–1.0, and then the overexpression of protein was induced by adding 0.3 mM IPTG at 18 °C for 12–16 h. The cultures were harvested by centrifuging at 4000× *g* for 10 min and resuspended in buffer A (50 mM HEPES pH 8.0, 1000 mM NaCl, 1 mM PMSF, 0.1% Triton X-100, 5% glycerol, 2 mM beta-mercaptoethanol). The cells were lysed by sonication, and lysates were clarified by centrifuging at 20,000× *g* for 45 min. The supernatant was then filtrated through a 0.45 μm filter membrane to remove cell debris and then applied to a Ni-chelating affinity column (GE Healthcare, Chicago, IL, USA). After the sample was loaded, the column was washed with buffer B (buffer A containing 20 mM imidazole), and the target protein was eluted with buffer C (buffer A containing 50 mM imidazole). TEV protease (1:10 weight ratio) was added to the eluent-containing protein including constructs WT NMAD-1A_21-263_ and the mut3-NMAD-1A_1-291_, then the His-Tag was removed overnight. The digestion was reloaded onto Ni-chelating affinity beads to remove His-Tag and His-Tagged TEV protease. The other WT constructs and the mut3-NMAD-1A_21-291_ kept their His-Tag. Then, 5 mL Hitrap Q (GE Healthcare) was used for further purification. Gel-filtration buffer for NMAD-1A_21-263_ (25 mM Tris pH 8.0, 100 mM NaCl, 5 mM beta-mercaptoethanol, 5% *v*/*v* glycerol) was used for further purification by size exclusion chromatography (GE Healthcare). The mut3 NMAD-1A_21-291_ and the mut3 NMAD-1A_1-291_ with 21 bp 5’-overhang dsDNA (5’-GCAGCAACAGAAGAGGATCTCA-3’, 5’-CTGAGATCCTCTTCTGTTGCTG-3’) were incubated overnight in gel-filtration buffer. In the next step, buffer (25 mM HEPES pH 8.0, 100 mM NaCl, 5 mM beta-mercaptoethanol, 10 mM α-KG, 0.5 mM MnCl_2_) was used for further purification by size exclusion chromatography (GE Healthcare). Fractions were analyzed by SDS-PAGE and the target protein was combined and concentrated to 20 mg/mL for crystallization.

### 5.2. In Vitro Demethylase Assays by a Methylation-Sensitive Restriction Digest Method

*C. elegans* NMAD-1A 6mA demethylation activity assays of ssDNA were modified from a prior publication [42]. First, 5 μM *C. elegans* NMAD-1A was incubated with 1 μM FAM-ssDNA_6mA1 (5’-FAM-GGATGCAAGCATCAGCAACAGAAGAGG (6mA)-TCTCAGGTGCAGCGC-3’, Invitrogen, Waltham, MA, USA) in reaction buffer containing 1 mM L-ascorbic acid, 0.2 mM (NH_4_)_2_Fe(SO_4_)_2_, 5 mM α-KG, 50 mM HEPES pH 8.0, 50 mM KCl, 12 mM MgCl_2_ at 37 °C for various times. The reactions were stopped by heating to 95 °C after adding 1.15 equivalent of ssDNA2 (5’-GCGCTGCACCTGAGATCCTCTTCTGTTGCTG-ATGCTTGCATCC-3’, Invitrogen) and *Dpn* II buffer. The samples were slowly cooled to room temperature for DNA annealing. *Dpn* II (0.5 U/mL) was added to the sample and kept at 37 °C for 1 h. All samples were analyzed by 6.7% native polyacrylamide gel (acrylamide: bisacrylamide = 19:1) and DNA was analyzed with a ChemiDoc^TM^ MP Imaging System (BIO-RAD, Hercules, CA, USA) at the wavelength of 488 nm. Quantitative calculation of gray value in gel images was integrated by using Image Lab 6.0 (BIO-RAD) software; then, the statistical analyses were carried out using OriginPro 2023b SR1 10.0.5.157 (Learning Edition) software. To show the demethylation results clearly, the (product integrations)/(substrate plus product integrations) were regarded as conversions.

For Bubble DNA and Bulge DNA 6mA demethylation assays, FAM-ssDNA_6mA1 was annealed with different paired DNA (Figures S1 and S2) to form Bubble/Bulge DNA by gradient annealing procedures at first. The paired primers were mixed together in a ratio of 1:1 and put into the PCR machine. The temperature was 95 °C for 5 min, then lowered to 80 °C for 2 min. After that, the temperature was lowered by 3 °C every 2 min until it reached 4 °C and then lasted for 10 min. Then, the enzymatic activity was determined by the same method as ssDNA, except, for the gradient annealing method after adding 1.1 equivalent of ssDNA2, ssDNA2 was paired with FAM-ssDNA_6mA1 to ensure the elimination of mismatched DNA. The temperature was 95 °C for 5 min, then lowered to 85 °C for 2 min. After that, the temperature was lowered by 3 °C every 2 min until it reached 4 °C and then kept for 10 min. Note that due to the excess of ssDNA2, all the FAM-ssDNA_6mA1 and its demethylated product were paired completely with ssDNA2 except for Bubble1 and Bulge1.

### 5.3. Crystallization and Data Collection

To obtain the *C. elegans* NMAD-1A crystals, many different truncations or mutants and extensive crystallization screens were performed at both 18 °C and 4 °C using many commercial kits. The *C. elegans* WT NMAD-1A_21-263_ formed sea urchin-shaped crystals at 4 °C in the reservoir solution of 25% PEG 3350, 200 mM CH_3_COONH_4_, 100 mM Bis-Tris pH 6.5 firstly. After a lot of crystallization optimizations, the high-quality crystals (Figure S4B) were obtained by seeding in a solution of 15.4% PEG 3350, 9% PEG 4000, 140 mM CH_3_COONH_4_, 60 mM (NH_4_)_2_SO_4_, 70 mM Bis-Tris pH 6.5, 30 mM Na cacodylate pH 6.5, 5% glycerol for two months at 4 °C. Previous studies found that 6mA was significantly enriched in AGAA and GAGG motifs in *C. elegans*, AGAAGAGGA motif in mice, and [G/C] AGG [C/T] motif in humans [12,73,76]. Therefore, we designed several nucleotide types used for the mut3 E109K/Q112K/Q114K NMAD-1A protein crystallization, containing a 5′-AGAAGAGGA-3′ motif in a single or double strand (Table S1).

The mut3 NMAD-1A_21-291_-Mn^2+^ plate crystals were obtained at 4 °C in a reservoir solution of 20% PEG 4000, 100 mM Na cacodylate pH 5.6 first. The mut3 NMAD-1A_21-291_-Mn^2+^ formed diamond-shaped crystals (Figure S4E) at 16 °C in a reservoir solution of 22% PEG 4000, 100 mM Na cacodylate pH 5.6 after optimizing. The mut3 NMAD-1A_1-291_-Mn^2+^-α-KG formed diamond-shaped crystals (Figure S4H) at 16 °C in a reservoir solution of 25% PEG 4000, 100 mM MES pH 5.6. To collect the data, the crystals were gradually transferred to a cryo-buffer (25% glycerol was added to the reservoir buffer) and flash-frozen in liquid N_2_. The data were collected on the beamlines BL02U1, BL18U1, and BL10U2 of the Shanghai Synchrotron Radiation Facility. All data were integrated and scaled with the HKL2000 suite of programs [77]. Due to the poor quality of NMAD-1A_21-263_ and mut3 NMAD-1A_21-291_ data, we collected at least 3 complete data and then merged them together to obtain standard data parameters such as completeness and redundancy. Data collection and processing statistics are shown in Table S2.

### 5.4. Structure Determination

The WT construct NMAD-1A_21-263_ was determined by molecular replacement using a predicted model by RaptorX v1.2.1 [78] as an MR model. The final structure was refined to an *R*_work_ of 24.9% and an *R*_free_ of 27.3% using the program COOT [79] followed by PHENIX software (version 1.18.2-3874) packages [80] for refinement many times. The space group of crystal NMAD-1A_21-263_ was *P*2_1_. Each cell contained one protein molecule. The mut3 NMAD-1A_21-291_-Mn^2+^ and mut3 NMAD-1A_1-291_-Mn^2+^-α-KG were determined by molecular replacement using the NMAD-1A_21-263_ structure as a model. Both the space groups of mut3 NMAD-1A_21-291_-Mn^2+^ and mut3 NMAD-1A_1-291_-Mn^2+^-α-KG crystals were *C*2. And each cell contained four protein molecules. The final structure of the mut3-NMAD-1A_21-291_-Mn^2+^ was refined to an *R*_work_ of 26.0% and an *R*_free_ of 28.2%. The final structure of the mut3 NMAD-1A_1-291_-Mn^2+^-α-KG was refined to an *R*_work_ of 27.5% and an *R*_free_ of 29.6%. All the figures in this article showing molecular structure were made by PyMOL v2.5.7 [81].

The atomic coordinates and structure factors of the WT NMAD-1A_21-263_-SO_4_^2−^, the mut3 NMAD-1A_21-291_-Mn^2+^, and mut3 NMAD-1A_1-291_-Mn^2+^-α-KG complex have been deposited in the Protein Data Bank under the accession codes 8HAZ, 8HBB, and 8HB2.

### 5.5. Microscale Thermophoresis

Microscale thermophoresis (MST) [82] was used to measure the dissociation constant for the interaction of different WT NMAD-1A constructs or mutants with α-KG cofactor. To measure the binding affinities between α-KG and NMAD-1A, WT NMAD-1A constructs or mutants were labeled using His-Tag labeling kit RED-tris-NTA. First, 5 μL of 10 μM labeled protein in buffer containing 30 mM HEPES pH 7.5, 150 mM KCl, 0.3% NP-40, 0.15% Triton X-100, 0.3% PEG 8000, 5 μM MnCl_2_ was mixed with 5 μL α-KG at various concentrations. After incubation for 60 min at room temperature, the samples were loaded into capillaries. Thermophoresis was measured on a Monolith NT.115 (NanoTemper, San Francisco, CA, USA) at 22 °C for 30 s with 40% infrared laser power. Datasets were combined and analyzed using the MO-Affinity analysis software (version 3.0) [83].

### 5.6. Small-Angle X-ray Scattering (SAXS)

To better investigate the natural oligomeric states of NMAD-1A in solution, we performed small-angle X-ray scattering measurements on beamline BL19U2 at the Shanghai Synchrotron Radiation Facility. Proteins, including WT full-length NMAD-1A_1-291_, WT construct NMAD-1A_21-263_, the mut3-NMAD-1A_1-291_, and mut3-NMAD-1A_21-291_, were purified by gel filtration in a buffer containing 25 mM HEPES pH 7.5, 100 mM NaCl, 5 mM β-mercaptoethanol. Various concentrations of proteins ranging from 1 to 4 mg/mL were used. Measurements were carried out at 10 °C. Then, 2D individual data were processed by RAW to 1D data [72,84,85,86]. The control buffer was measured before and after each sample measurement. GNOM [87] provided the pair distribution function P(r) of the particle, the maximum size d_max_, and the Porod volume. The 20 individual ab initio reconstructions were generated with DAMMIN [88], averaged using DAMAVER [89], and aligned using SUPCOMB [90]. The structures were visualized using PyMOL v2.5.7.

### 5.7. Electrophoretic Mobility Shift Assay

Electrophoretic mobility shift assay (EMSA) was performed to detect the nucleosome-binding ability of NMAD-1A or different constructs [91]. First, 1.0 μM to 2.0 μM concentration NMAD-1A protein was incubated with 0.5 μM nucleosomes in a 20 μL reaction mixture containing 30 mM HEPES pH 7.5, 150 mM KCl, 200 μM α-KG, 5 μM MnCl_2_ for 30 min at 4 °C. After incubation, 5 μL 30% glycerol was added to each sample and 5 μL of the mixture was then separated on a 6.7% native polyacrylamide gel (acrylamide: bisacrylamide = 29:1) in 1×TB (45 mM Tris pH 8.3, 45 mM boric acid) at 175 V for 45 min at 4 °C. Nucleosomes and nucleosome–DNA complexes were visualized by SYBR Red (Molecular Probes EMSA kit, Invitrogen, Shanghai, China) scanned with a ChemiDoc MP Imaging System (BIO-RAD, Carlsbad, CA, USA).

## Data Availability

Atomic coordinates and structure factors for the reported crystal structures have been deposited with the Protein Data Bank under accession numbers 8HAZ, 8HBB, and 8HB2.

## Supplementary Materials

The following supporting information can be downloaded at: https://www.mdpi.com/article/10.3390/ijms25020686/s1. References [92,93,94,95] are cited in the supplementary materials.

## Abbreviations

The abbreviations used are: α-KG, α-ketoglutarate; AlkB, alpha-ketoglutarate-dependent dioxygenase; ALKBH, AlkB homolog; ANOVA, analysis of variance; DSBH, double-stranded β-helix; Flips (Flip1, Flip2), the components of nucleotide-recognition lids which can recognize nucleic acids and flip the methylated lesion; MST, microscale thermophoresis; NRLs, nucleotide-recognition lids; TEV, tobacco etch virus; IPTG, isopropyl-β-D-thiogalactopyranoside; NTE, amino-terminal extension; NOG, N-oxalylglycine; ZFD, zinc finger domain; CTD, carboxy-terminal domain; PMSF, phenylmethylsulfonyl fluoride; PDB, Protein Data Bank; PEG, polyethylene glycol; Tris, trishydroxymethylaminomethane; HEPES, N-(2-hydroxyethyl) piperazine-N′-(2-ethanesulfonic acid); FAM, carboxy-fluorescein; FPLC, fast protein liquid chromatography; FTO, fat mass and obesity-associated protein; PCR, polymerase chain reaction; 6mA, (DNA)*N*^6^-methyladenosine; 1mA, (DNA) *N*^1^-methyladenosine; m^6^A, (RNA) *N*^6^-methyladenosine; r.m.s.d., root mean squared deviation.
