# Supplementary material for: Structural Basis of Nucleic Acid Recognition and 6mA Demethylation by Caenorhabditis elegans NMAD-1A
Source: Int J Mol Sci. 2024 Jan 5;25(2):686. doi: 10.3390/ijms25020686 (PMC10815869; doi:10.3390/ijms25020686)
Supplement: Supplementary file 1 [file ijms-25-00686-s001.zip › ijms-2789806-supplementary.pdf]

Supplementary Information

Structural basis of nucleic acid recognition and 6mA demethylation by *Caenorhabditis elegans* NMAD-1A

Guohui Shang[1], Meiting Yang[1], Min Li[2], Lulu Ma[1], Yunlong Liu[3], Jun Ma[1], Yiyun Chen[4], Xue Wang[1], Shilong Fan[2], Mengjia Xie[1], Wei Wu[1], Shaodong Dai[5], and Zhongzhou Chen[1,*]

Name	Sequence	6mA: ★ Base: ●
ssDNA	5'-FAM-GGATGCAAGCATCAGCAACAGAAGAGG(6mA)TCTCAGGTGCAGCGC-3'	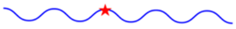
dsDNA	5'-FAM-GGATGCAAGCATCAGCAACAGAAGAGG(6mA)TCTCAGGTGCAGCGC-3'<br>3'-CCTACGTTCGTAGTCGTTGTCTTCTCC T AGAGTCCACGTCGCG-5'	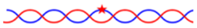
Bubble1	5'-FAM-GGATGCAAGCATCAGCAACAGAAGAGG(6mA)TCTCAGGTGCAGCGC-3'<br>3'-CCTACGTTCGTAGTCGTTGTCTTCTCC G AGAGTCCACGTCGCG-5'	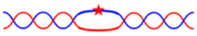
Bubble2	5'-FAM-GGATGCAAGCATCAGCAACAGAAGAGG(6mA)TCTCAGGTGCAGCGC-3'<br>3'-CCTACGTTCGTAGTCGTTGTCTTCTCC G CGAGTCCACGTCGCG-5'	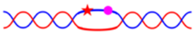
Bubble3	5'-FAM-GGATGCAAGCATCAGCAACAGAAGAGG(6mA)TCTCAGGTGCAGCGC-3'<br>3'-CCTACGTTCGTAGTCGTTGTCTTCTCA G CGAGTCCACGTCGCG-5'	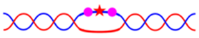
Bubble4	5'-FAM-GGATGCAAGCATCAGCAACAGAAGAGG(6mA)TCTCAGGTGCAGCGC-3'<br>3'-CCTACGTTCGTAGTCGTTGTCTTCTCA G CTAGTCCACGTCGCG-5'	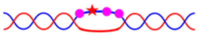
Bubble5	5'-FAM-GGATGCAAGCATCAGCAACAGAAGAGG(6mA)TCTCAGGTGCAGCGC-3'<br>3'-CCTACGTTCGTAGTCGTTGTCTTCTTA G CTAGTCCACGTCGCG-5'	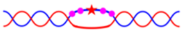
Bubble6	5'-FAM-GGATGCAAGCATCAGCAACAGAAGAGG(6mA)TCTCAGGTGCAGCGC-3'<br>3'-CCTACGTTCGTAGTCGTTGTCTTCTTA G CTGGTCCACGTCGCG-5'	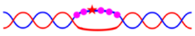
Bubble7	5'-FAM-GGATGCAAGCATCAGCAACAGAAGAGG(6mA)TCTCAGGTGCAGCGC-3'<br>3'-CCTACGTTCGTAGTCGTTGTCTTCATA G CTGGTCCACGTCGCG-5'	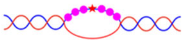
Bubble8	5'-FAM-GGATGCAAGCATCAGCAACAGAAGAGG(6mA)TCTCAGGTGCAGCGC-3'<br>3'-CCTACGTTCGTAGTCGTTGTCTTCATA G CTGTTCCACGTCGCG-5'	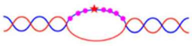
Bulge1	5'-FAM-GGATGCAAGCATCAGCAACAGAAGAGG(6mA)TCTCAGGTGCAGCGC-3<br>3'-CCTACGTTCGTAGTCGTTGTCTTCTCC-----AGAGTCCACGTCGCG-5'	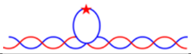
Bulge2	5'-FAM-GGATGCAAGCATCAGCAACAGAAGAGG(6mA)TCTCAGGTGCAGCGC-3'<br>3'-CCTACGTTCGTAGTCGTTGTCTTCTC------AGAGTCCACGTCGCG-5'	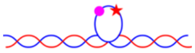
Bulge3	5'-FAM-GGATGCAAGCATCAGCAACAGAAGAGG(6mA)TCTCAGGTGCAGCGC-3'<br>3'-CCTACGTTCGTAGTCGTTGTCTTCTC-------GAGTCCACGTCGCG-5'	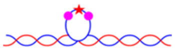
Bulge4	5'-FAM-GGATGCAAGCATCAGCAACAGAAGAGG(6mA)TCTCAGGTGCAGCGC-3'<br>3'-CCTACGTTCGTAGTCGTTGTCTTCTC--------AGTCCACGTCGCG-5'	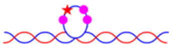
Bulge5	5'-FAM-GGATGCAAGCATCAGCAACAGAAGAGG(6mA)TCTCAGGTGCAGCGC-3'<br>3'-CCTACGTTCGTAGTCGTTGTCTTCT---------AGTCCACGTCGCG-5'	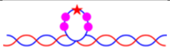
Bulge6	5'-FAM-GGATGCAAGCATCAGCAACAGAAGAGG(6mA)TCTCAGGTGCAGCGC-3'<br>3'-CCTACGTTCGTAGTCGTTGTCTTCT----------GTCCACGTCGCG-5'	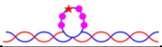
Bulge7	5'-FAM-GGATGCAAGCATCAGCAACAGAAGAGG(6mA)TCTCAGGTGCAGCGC-3'<br>3'-CCTACGTTCGTAGTCGTTGTCTTC-----------GTCCACGTCGCG-5'	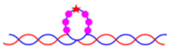
Bulge8	5'-FAM-GGATGCAAGCATCAGCAACAGAAGAGG(6mA)TCTCAGGTGCAGCGC-3'<br>3'-CCTACGTTCGTAGTCGTTGTCTTC------------TCCACGTCGCG-5'	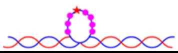

Figure S1. Schematic diagram of 6mA ssDNA, dsDNA, Bubble, and Bulge DNA.

Name	Sequence	6mA: ★ Base: ●
**Bulge6-1**	5'-FAM-GGATGCAAGCATCAGCAACAGAAGAGG(6mA)TCTCAGGTGCAGCGC-3'<br>3'-CCTACGTTCGTAGTCGTTGTCTTCTCC----------CCACGTCGCG-5'	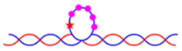
**Bulge6-2**	5'-FAM-GGATGCAAGCATCAGCAACAGAAGAGG(6mA)TCTCAGGTGCAGCGC-3'<br>3'-CCTACGTTCGTAGTCGTTGTCTTCTC----------TCCACGTCGCG-5'	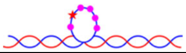
**Bulge6-3**	5'-FAM-GGATGCAAGCATCAGCAACAGAAGAGG(6mA)TCTCAGGTGCAGCGC-3'<br>3'-CCTACGTTCGTAGTCGTTGTCTTCT----------GTCCACGTCGCG-5	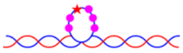
**Bulge6-4**	5'-FAM-GGATGCAAGCATCAGCAACAGAAGAGG(6mA)TCTCAGGTGCAGCGC-3'<br>3'-CCTACGTTCGTAGTCGTTGTCTTC----------AGTCCACGTCGCG-5'	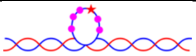
**Bulge6-5**	5'-FAM-GGATGCAAGCATCAGCAACAGAAGAGG(6mA)TCTCAGGTGCAGCGC-3'<br>3'-CCTACGTTCGTAGTCGTTGTCTT----------GAGTCCACGTCGCG-5'	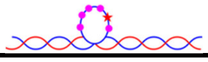
**Bulge6-6**	5'-FAM-GGATGCAAGCATCAGCAACAGAAGAGG(6mA)TCTCAGGTGCAGCGC-3'<br>3'-CCTACGTTCGTAGTCGTTGTCT----------AGAGTCCACGTCGCG-5'	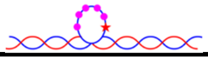

Figure S2. Schematic diagram of 6mA Bulge6 DNA.

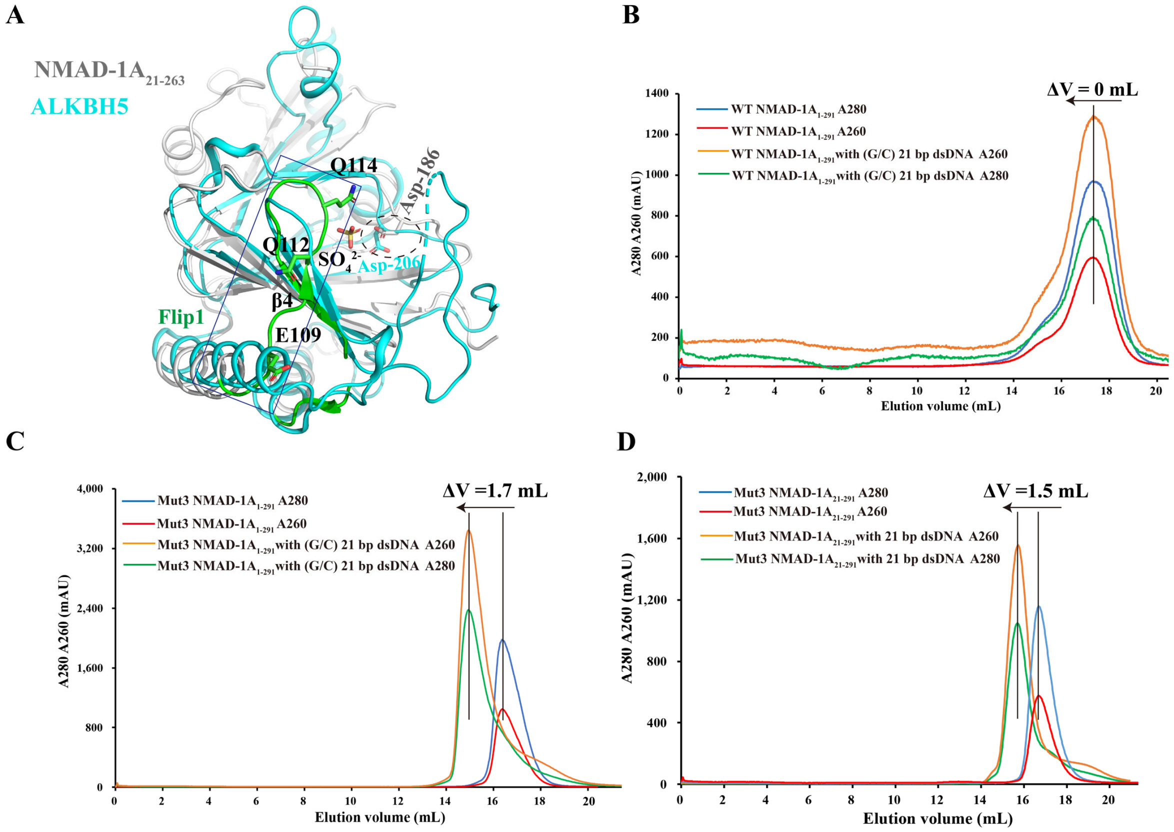

Figure S3. The structure comparisons of NMAD-1A (light grey) and ALKBH5 (cyan; PDB: 4NRM), especially the conformation Asp-186 (NMAD-1A) and Asp-206 (ALKBH5) (A). The peak volume migration of WT NMAD-1A$_{1\text{-}291}$ (B), the mut3 NMAD-1A$_{1\text{-}291}$ (C)/ NMAD-1A$_{21\text{-}291}$ (D) and (G/C) 21bp dsDNA (Supplementary Table S1) complex compared with different NMAD-1A proteins for the SEC.

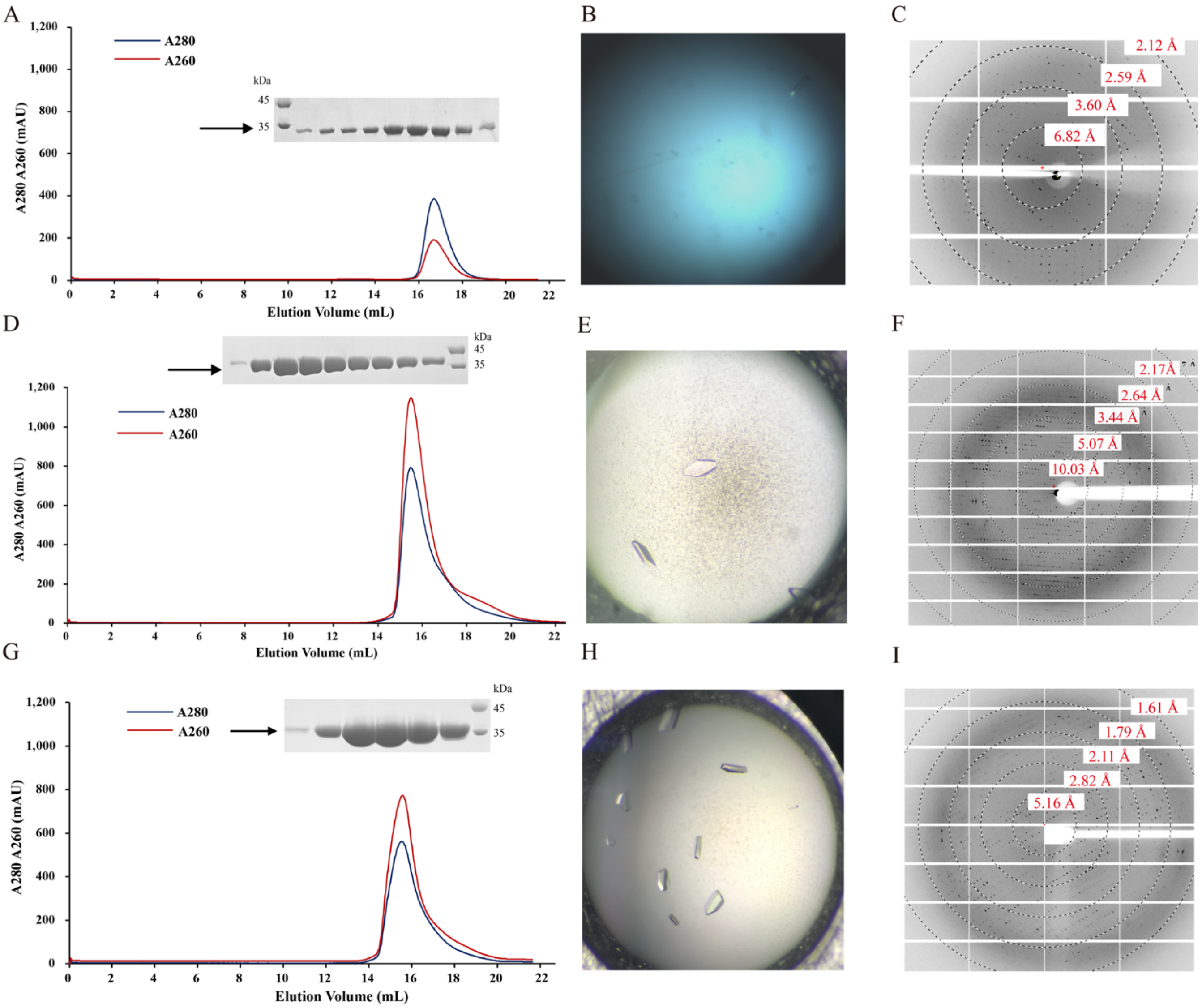

Figure S4. The size exclusion chromatogram (SEC), crystals, and X-ray diffraction patterns of WT NMAD-1A truncations and mutants. (A-C) The SEC, crystals, and X-ray diffraction patterns of WT NMAD-1A$_{21-263}$ from left to right. (D-F) The SEC, crystals, and X-ray diffraction patterns of the mut3 (E109K/Q112K/Q114K) NMAD-1A$_{21-291}$ with (G/C) 21 bp dsDNA (Table S1) from left to right. (G-I) The SEC, crystals, and X-ray diffraction patterns of the mut3 (E109K/Q112K/Q114K) NMAD-1A$_{1-291}$ with (G/C) 21 bp dsDNA (Table S1) from left to right.

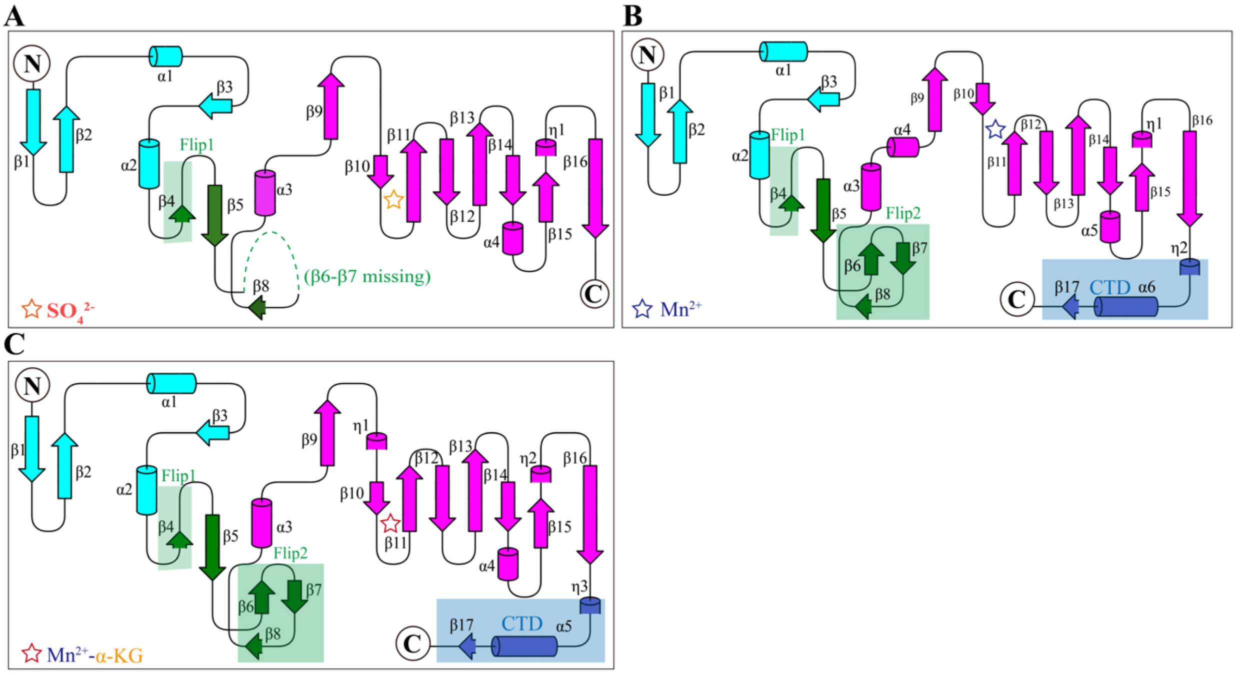

Figure S5. Topologies of NMAD-1A$_{21\text{-}263}$-SO_4^{2-} (A), the mut3-NMAD-1A$_{21\text{-}291}$-Mn^{2+} (B), and mut3-NMAD-1A$_{1\text{-}291}$-Mn^{2+}-α-KG (C). Cofactors are denoted by different color stars. The NTE, NRL, DSBH, and CTD are colored in cyan, green, magenta, and slate, respectively.

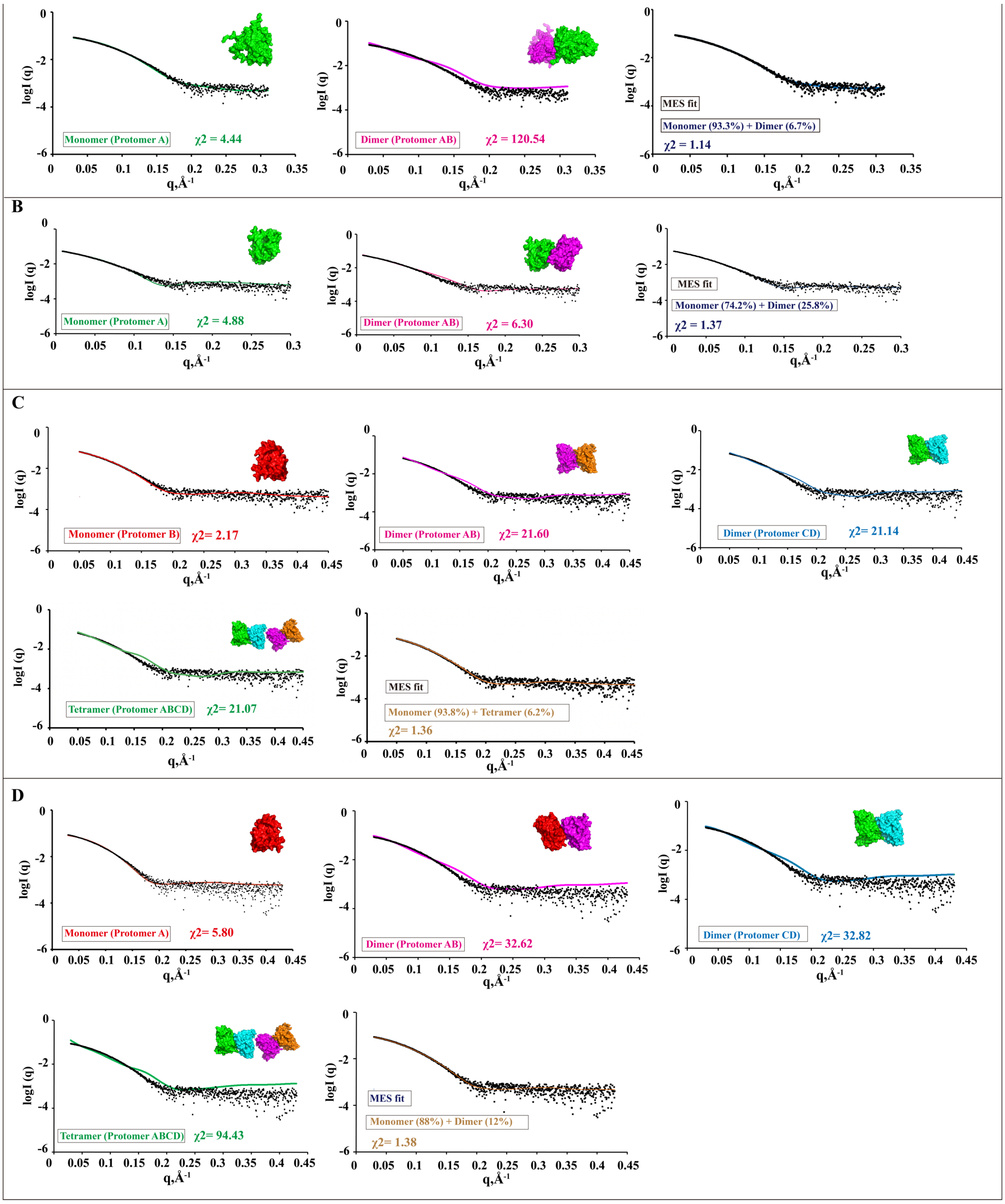

Figure S6. The oligomeric analysis of WT NMAD-1A, NMAD-1A constructs, and mutants. Oligomeric states of WT full-length NMAD-1A$_{1-291}$ (A) (with WT NMAD-1A$_{1-291}$ predicted from AlphaFold2 Protein Structure Database (ebi.ac.uk)), WT NMAD-1A$_{21-263}$ (B), the mut3-NMAD-1A$_{21-291}$ (C), and the mut3-NMAD-1A$_{1-291}$ (D). Experimental data are represented by black dots. The theoretical scattering curves of oligomers are colored differently.

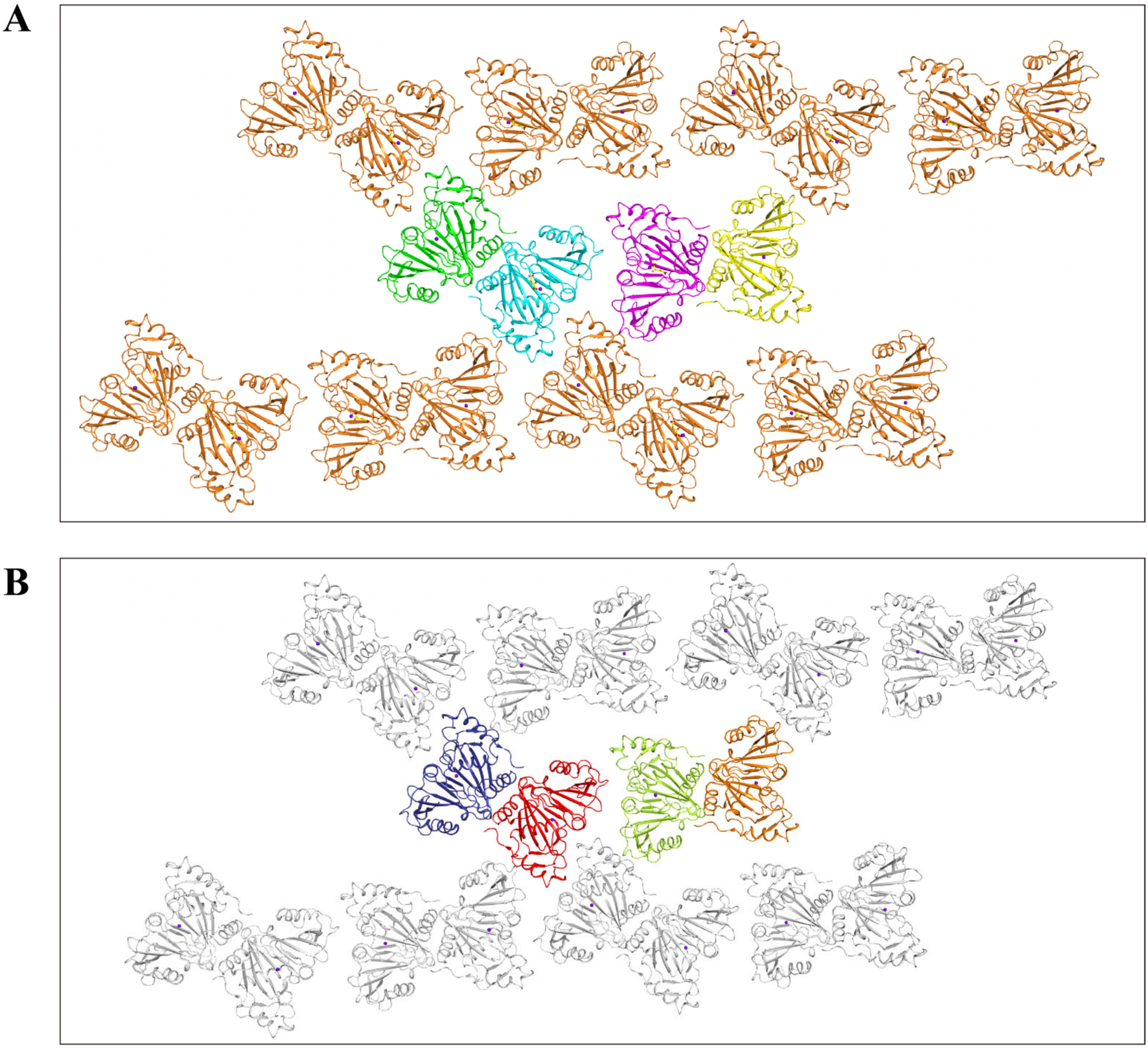

Figure S7. Crystal packing of the mut3-NMAD-1A$_{1-291}$-Mn^{2+}-α-KG (PDB: 8HB2) and the mut3-NMAD-1A$_{21-291}$-Mn^{2+} (PDB: 8HBB). (A) There are four copies of the mut3-NMAD-1A$_{1-291}$-Mn^{2+}-α-KG in each asymmetric unit. Mn^{2+}: purple-blue sphere, α-KG: yellow sticks, the mut3-NMAD-1A$_{1-291}$ protomers A, B, C, and D are shown as green, cyan, magenta, and yellow cartoon, respectively. (B) There are four copies of the mut3-NMAD-1A$_{21-291}$-Mn^{2+} in each asymmetric unit. Mn^{2+}: purple-blue sphere, the mut3-NMAD-1A$_{1-291}$ protomers A, B, C, and D are shown as red, deepblue, lemon, and orange cartoon, respectively.

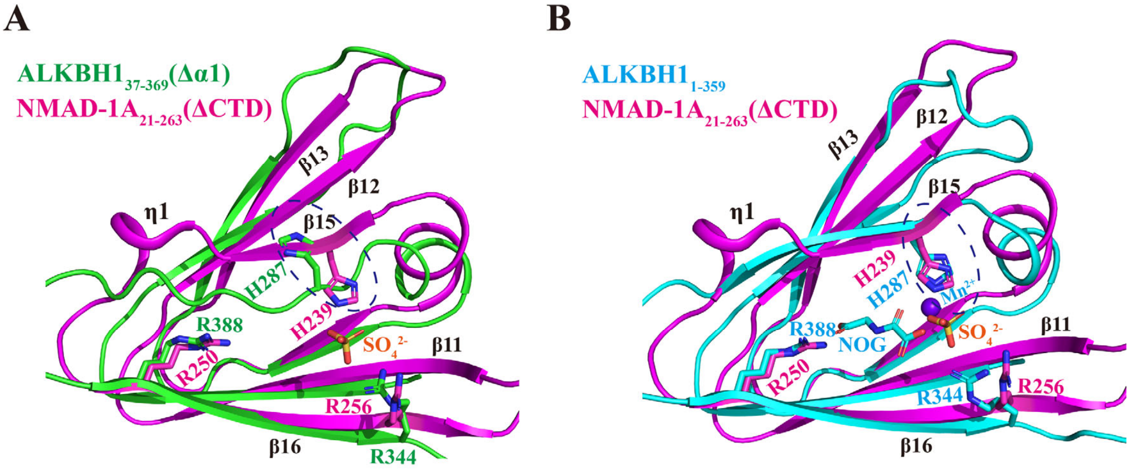

Figure S8. The structural comparisons of the catalytic center between NMAD-1A$_{21\text{-}263}$ (colored magenta) and ALKBH1$_{37\text{-}369}$ (colored green; PDB: 6IMA) [1] (A); NMAD-1A$_{21\text{-}263}$ and ALKBH1$_{1\text{-}359}$ (colored cyan; PDB: 6IMC) [1] (B). The conformation of His-287 (ALKBH1$_{37\text{-}369}$) is completely opposite compared with those of NMAD-1A$_{21\text{-}263}$ and ALKBH1$_{1\text{-}359}$ in blue dashed ellipse. Key residues involved in cofactor binding are shown as sticks. SO_4^{2-}, orange stick; Mn^{2+}, purple ball; α-KG, yellow stick.

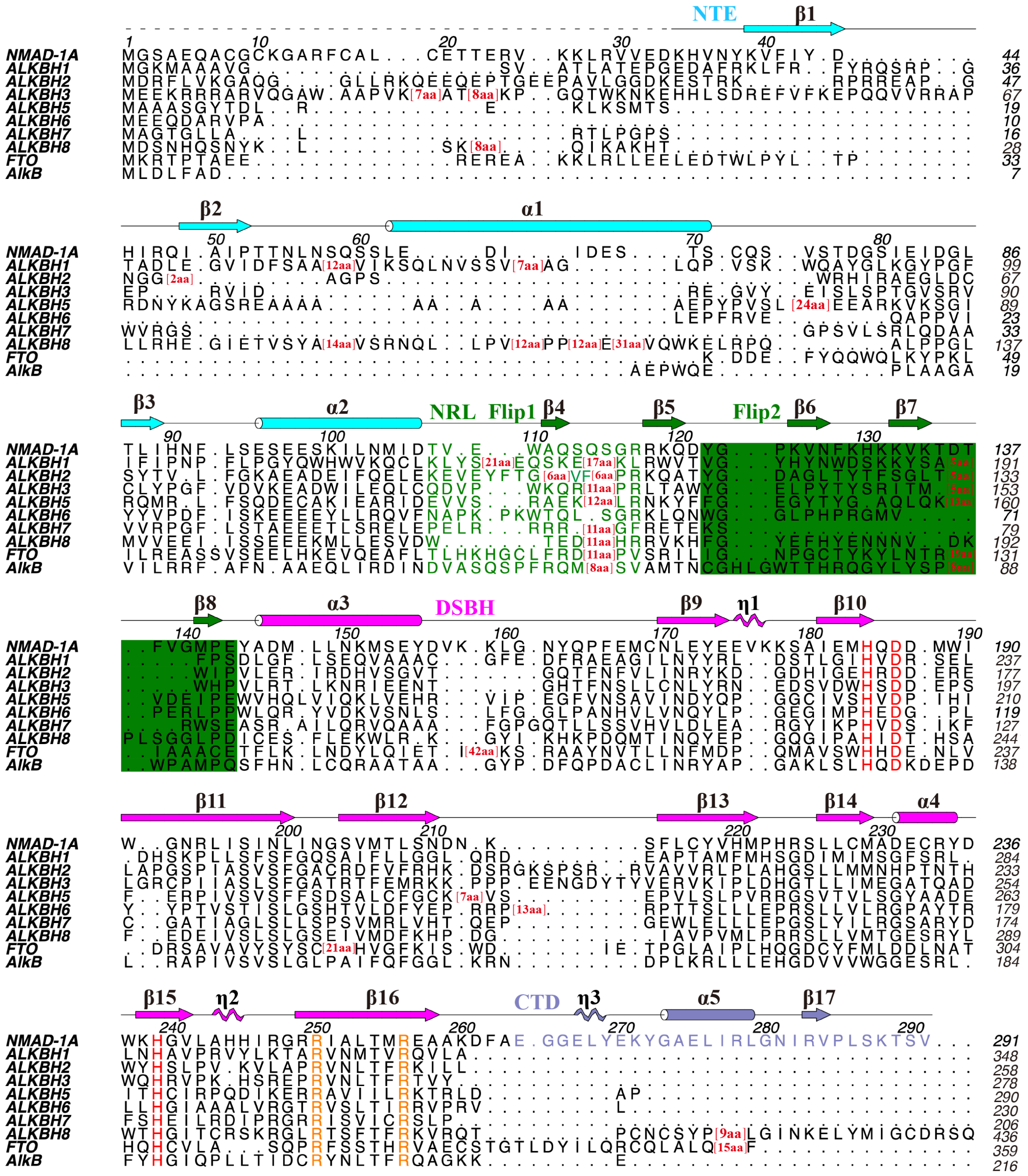

Figure S9. Structure-based sequence alignment of NMAD-1A and other AlkB family members. Structure-based sequence alignment of NMAD-1A with human AlkB homologs and *E. coli* AlkB using T-Coffee [2, 3]. Secondary structural elements of the mut3-NMAD-1A$_{1\text{-}291}$ structure are calculated using DSSP [4] and colored in cyan, green, magenta, and slate for NTE, NRL, DSBH, and CTD, respectively. The conserved residues (HxD…H) are colored in red, and residues (R…R) are colored in orange. The length of sequences omitted for the clarity of the presentation is shown in bracket.

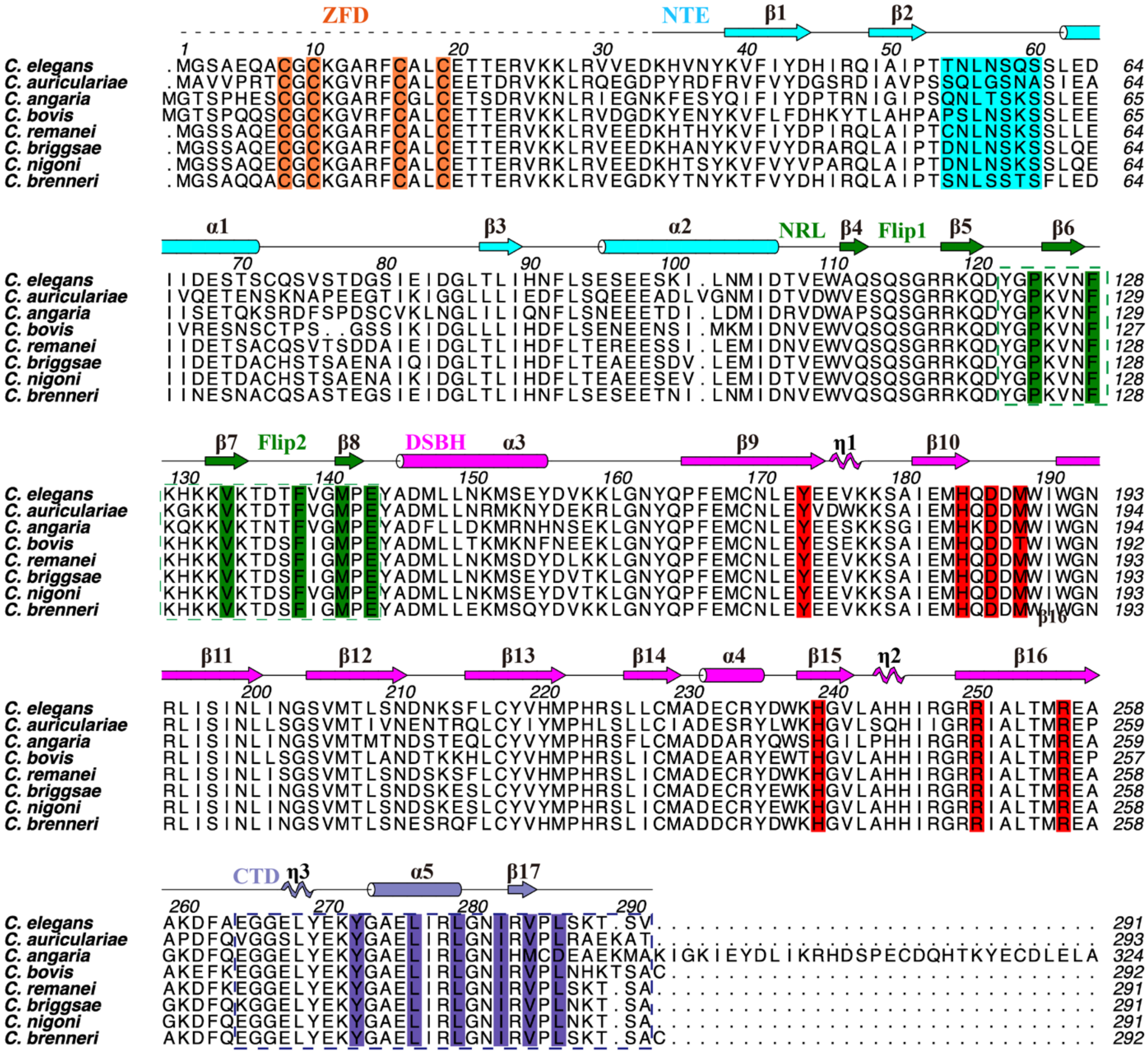

Figure S10. Structure-based sequence alignment of NMAD-1A orthologs. Mn^{2+} and α-KG binding residues are colored in red. The four cysteines forming the zinc finger domain (ZFD) are colored orange. The amino acid residues participating in the interaction between the CTD (slate dashed box) and the Flip2 region (green dashed box) are colored in slate and green, respectively. GenBank ID: *C. elegans*, NP_741141.1; *C. brenneri*, EGT39798.1; *C. remanei*, XP_003110949.1. *C. briggsae*, UMM22374.1; *C. auriculariae*, CAD6197438.1; *C. nigoni*, PIC42364.1; *C. angaria*, CAI5445535.1; *C. bovis*, CAB3402403.1.

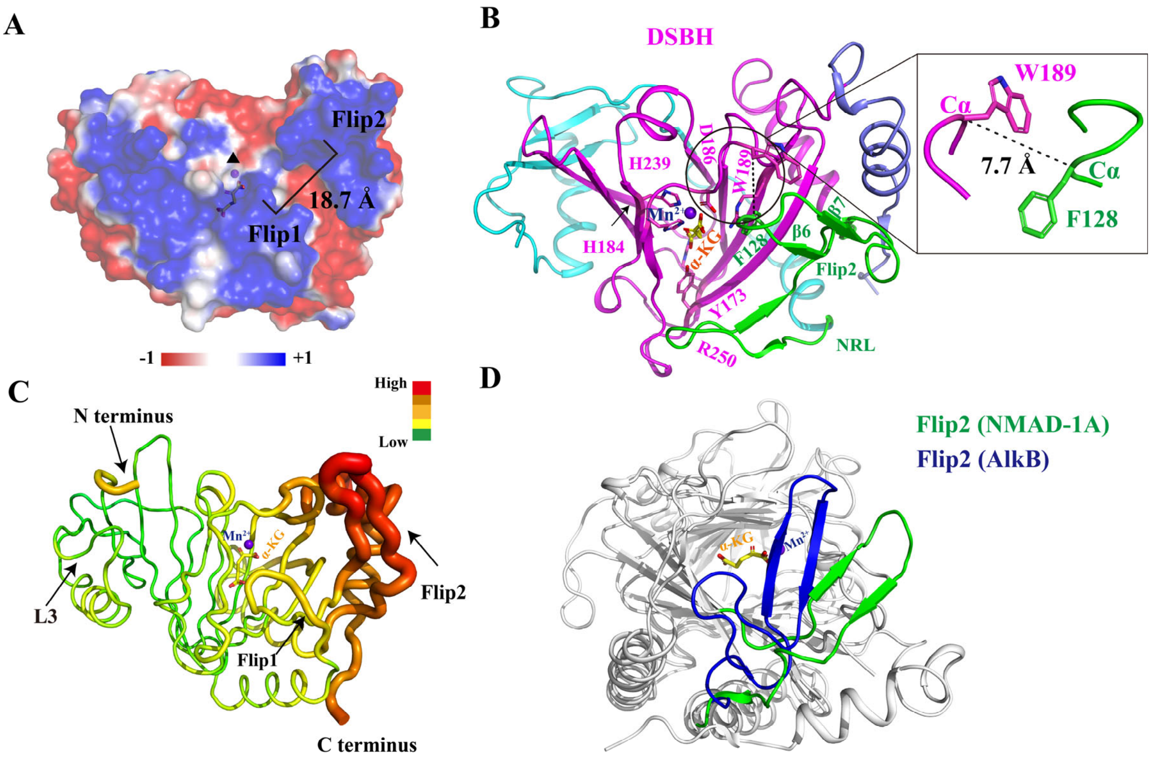

Figure S11. Global views of the electrostatic potential and the interacting pocket interface of NMAD-1A. (A) The surface potential of NMAD-1A. The width of the channel between the Flip1 and Flip2 of NMAD-1A (~18.7 Å) has enough space to accommodate the bulge region of Bulge DNA. (B) The distance between the Flip2 region and the opposing edge of the cleft of NMAD-1A was measured between F128 Cα and W189 Cα. (C) The temperature factor of the mut3 NMAD-1A$_{1\text{-}291}$-Mn^{2+}-α-KG structure. The temperature factors of the Flip2 are higher than those of the Flip1. (D) Representation of the Flip2 of NMAD-1A (green) and AlkB (blue). Mn^{2+}, a purple-blue sphere; α-KG, yellow sticks.

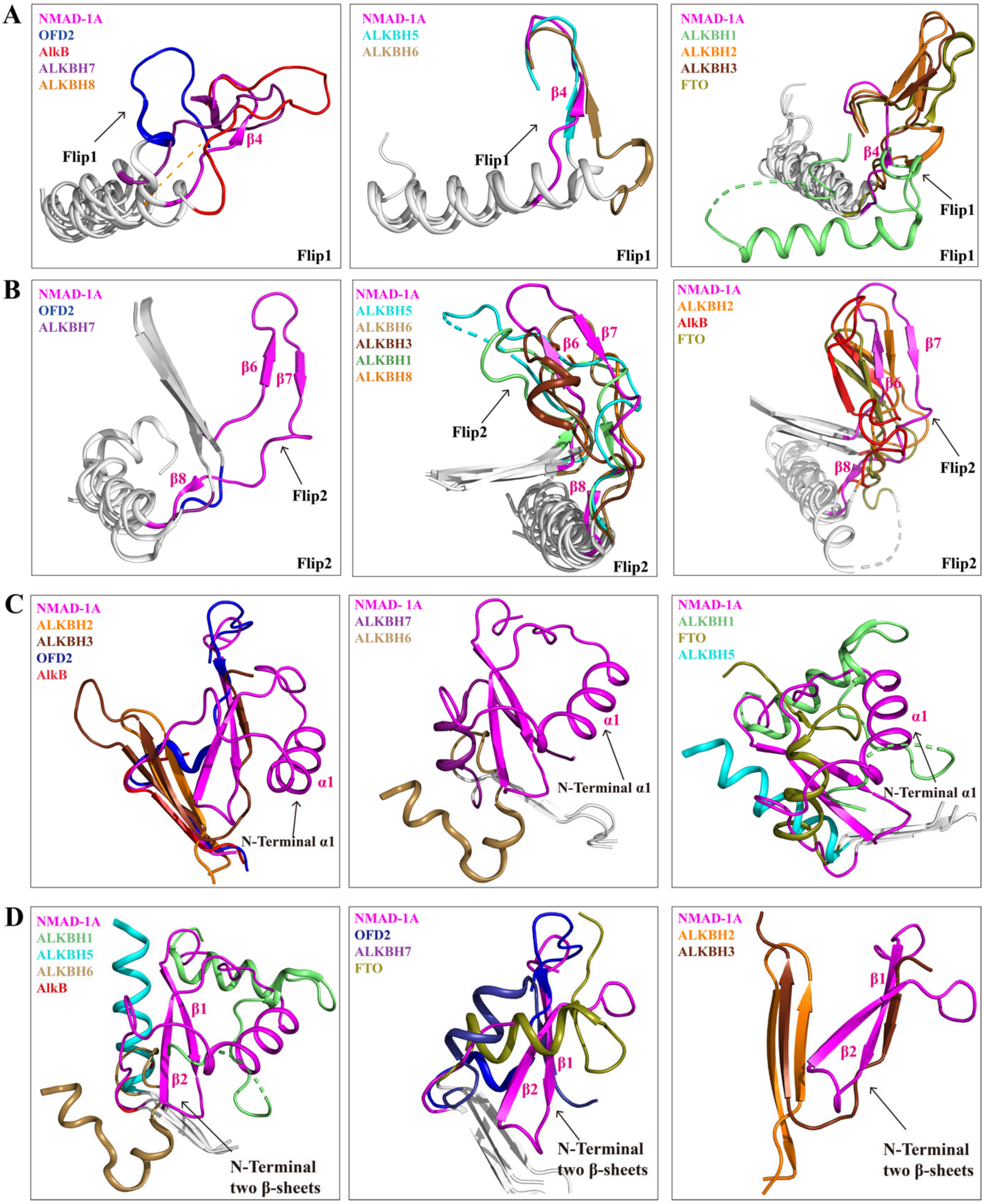

Figure S12. The differences of the secondary structures including the NTE and NRL between NMAD-1A and other AlkB family members. The differences of the Flip1 (A) and Flip2 (B) regions of NRL between NMAD-1A and other AlkB family members. PDB code: AlkB, 2FD8. ALKBH1, 6IE2. ALKBH2, 3S57. ALKBH3, 2IUW. ALKBH5, 4NRM. ALKBH6, 7VJV. ALKBH7, 4QKD. ALKBH8, 3THP. FTO 3LFM. OFD2, 5YLB. The differences of NTE

N-terminal $\alpha 1$ (C) and two β-sheets (D) between NMAD-1A and other AlkB family members. All members were shown in different colors.

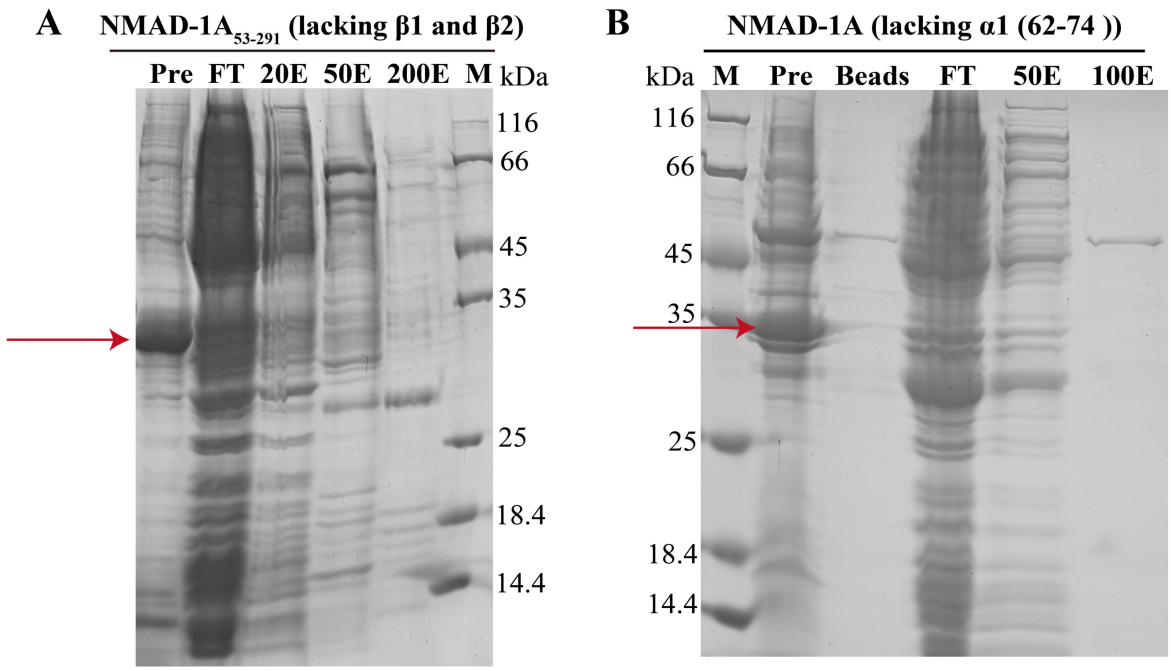

Figure S13. The NTE stabilizes the integrity of NMAD-1A. The eluted fractions of the lacking N-terminal β-sheets (β1, β2) construct NMAD-1A$_{53\text{-}291}$ (A) and lacking α-helix (α1) construct NMAD-1A (Δ62-74) (B) purified by Ni^{2+} affinity chromatography were detected by SDS-PAGE. Both were mainly expressed as the inclusion body in bacteria. Pre, the precipitate (inclusion body); FT, flow through; Beads, Ni-agarose resin; 20E, 50E, 100E, and 200E represent the fractions eluted at 20 mM, 50 mM, 100 mM, and 200 mM imidazole-containing buffer, respectively. The protein bands of interest (32~35 kDa) were indicated by the red arrows.

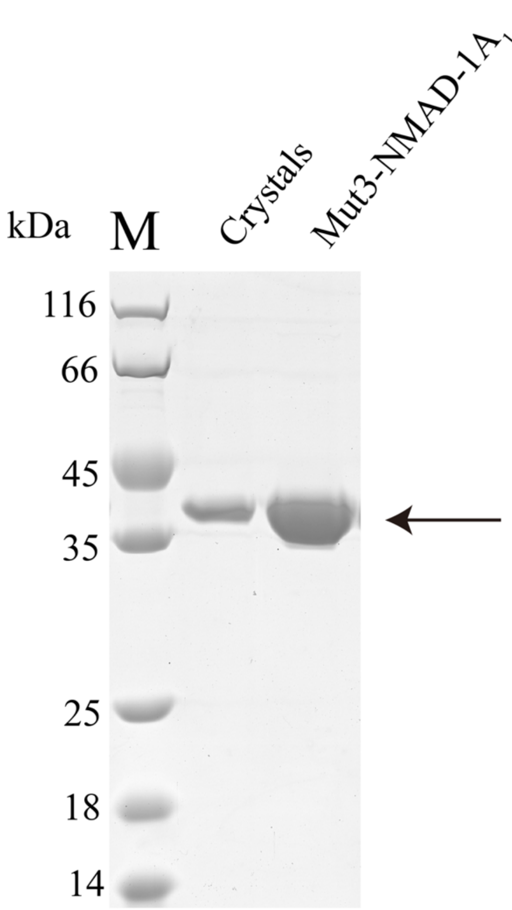

Figure S14. The mut3-NMAD-1A$_{1-291}$ proteins before and after crystallization. The molecular mass of these two proteins was equal (~35 kDa), suggesting that the missing ZFD in the mut3-NMAD-1A$_{1-291}$ structure owed to its flexibility.

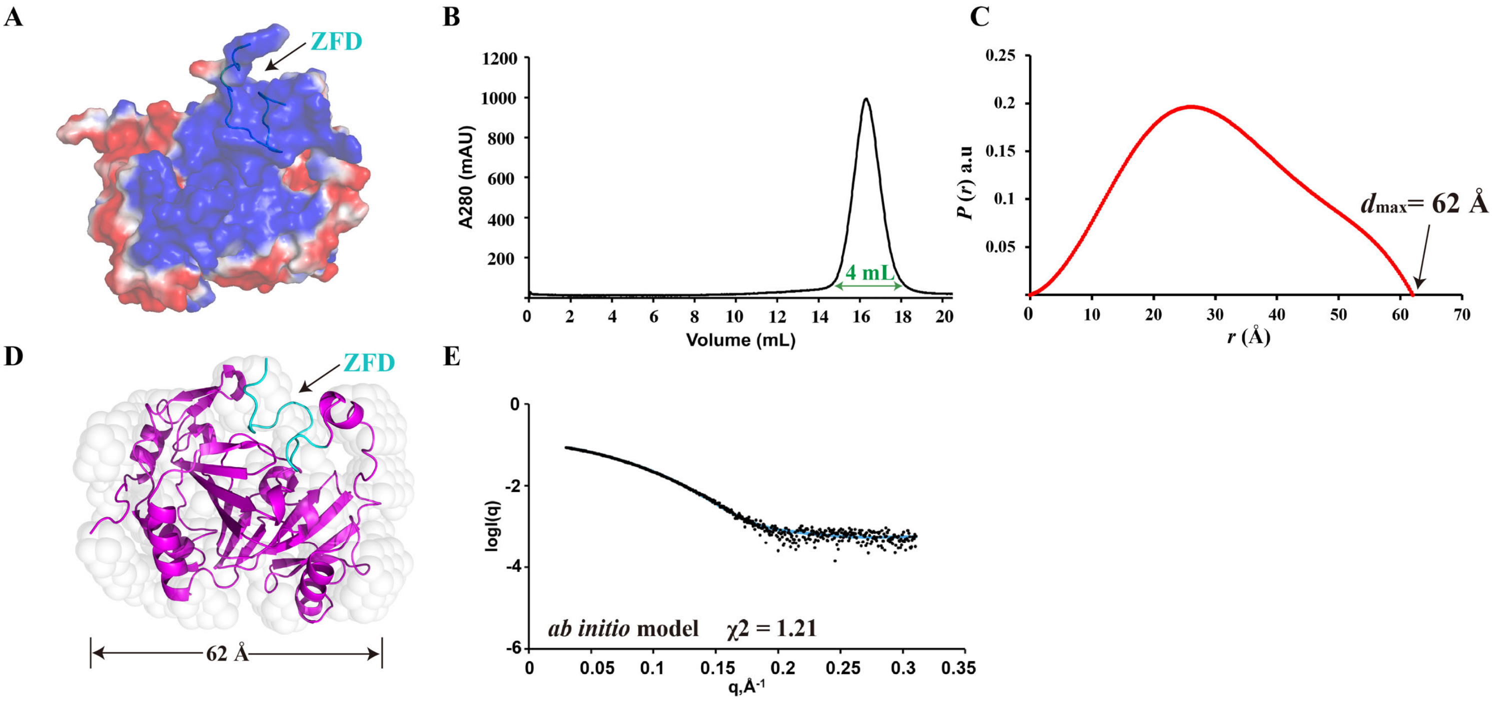

Figure S15. SAXS analysis of WT NMAD-1A$_{1\text{-}291}$ structure in solution. (A) Electrostatic surface presentations of WT NMAD-1A$_{1\text{-}291}$ predicted from AlphaFold2 Protein Structure Database (ebi.ac.uk), where red and blue describe negative and positive potentials, respectively. ZFD is shown as a loop cartoon and marked with a black arrow. (B) The SEC pattern of WT NMAD-1A$_{1\text{-}291}$. (C) Pair distance distribution $P(r)$ functions for NMAD-1A. (D) Superposition of low-resolution *ab initio* model and rigid body model. The *ab initio* model is shown as a light-gray surface representation. Molecule NMAD-1A is shown in magenta color. ZFD is shown as a cyan loop cartoon and marked with a black arrow. (E) Overlay of the experimental scattering profile with the back-calculated scattering profile from the DAMMIN model of NMAD-1A.

Table | S1. Nucleotide types used for NMAD-1A protein crystallization

Nucleotide types	Nucleotide base sequence
21 nt ssDNA	CAGCAACAGAAGAGGATCTCA
16 nt ssDNA	CAACAGAAGAGGATCT
(G/C) 12 bp dsDNA	**G**ACAGAAGAGGAT<br>TGTCTTCTCCTA**C**
(T/A) 15 bp dsDNA	**T**AAGAAGAGGATCTCA<br>TTCTTCTCCTAGAGT**A**
(T/A) 16 bp dsDNA	**T**ACAGAAGAGGATCTCA<br>TGTCTTCTCCTAGAGT**A**
(G/C) 18 bp dsDNA	**G**CAACAGAAGAGGATCTCA<br>GTTGTCTTCTCCTAGAGT**C**
(G/C) 21 bp dsDNA	**G**CAGCAACAGAAGAGGATCTCA<br>GTCGTTGTCTTCTCCTAGAGT**C**

Table | S2. Data collection and refinement statistics of NMAD-1A

	NMAD-1A$_{21\text{-}263}$-SO_4^{2-}	mut3-NMAD-1A$_{21\text{-}291}$-Mn^{2+}	mut3-NMAD-1A$_{1\text{-}291}$-Mn^{2+}-α-KG
**Data collection**			
Wavelength (Å)	0.9791	0.9791	0.9791
Space group	$P2_1$	$C2$	$C2$
Cell dimensions			
a, *b*, *c* (Å)	44.22, 48.53, 51.88	183.18, 75.33, 118.18	180.24, 75.39, 117.22
α, β, γ (˚)	90, 104.4, 90	90, 113.2, 90	90, 112.5, 90
Resolution (Å) [a]	50-2.70 (2.75-2.70)	50-3.10 (3.15-3.10)	30-3.06 (3.16-3.06)
R_{merge} (%)	9.3 (20.6)	8.4 (28.0)	9.8 (60.7)
I/σ	9.7 (3.6)	14.8 (4.1)	10.5 (2.6)
Completeness (%)	90.3 (82.6)	90.9 (76.9)	95.7 (99.9)
Total No. of reflections	19006	141160	116139
Unique reflections	6106	26900	26508
Redundancy	3.4 (2.4)	5.8 (4.5)	4.4 (3.6)
**Refinement**			
Resolution (Å)	50.0-2.70	50-3.10	30-3.06
No. of reflections	5265	22169	26487
R_{work}/R_{free} (%)	24.9/27.3	26.0/28.2	27.5/29.6
No. of atoms			
Protein	1720	7675	7919
Ligand/ion	5	9	24
Water	73	9	29
Average *B*-factors (Å^2) [b]			
Protein	45.88	49.78	64.90
Ligand/ions	45.68	46.66	58.91
Water	45.74	47.41	39.89
rms deviations			
Bond lengths (Å)	0.009	0.012	0.010
Bond angles (°)	0.989	1.657	1.382
Ramachandran plot (%)[c]	97.6/2.4/0	98.2/1.7/0.1	98.2/1.8/0

[a]Statistics for the highest resolution shell.

[b]Average *B*-factors were calculated by PHENIX Refinement of grouped *B*-factors (one per residue instead of one per atom).

[c]Residues in favoured, allowed, and outlier regions of the Ramachandran plot, respectively.

Table | S3. Statistics of SAXS analysis of NMAD-1A

Data-collection parameters	NMAD-1A$_{21-263}$	mut3-NMAD-1A$_{1-291}$	mut3-NMAD-1A$_{21-291}$	WT-NMAD-1A$_{1-291}$
**Instrument**				
Wavelength (Å)	1.03	1.03	1.03	1.03
Exposure time	1 sec	1 sec	1 sec	1 sec
Beam size (μm)	320×43	320×43	320×43	320×43
Temperature	283 K	283 K	283 K	283 K
Camera length (m)	2.68	2.68	2.68	2.68
Sample temperature (℃)	10.0	10.0	10.0	10.0
**Molecular mass**				
Loading concentration (mg ml^{-1})	2.0	2.0	2.0	1.0
M from chemical composition (Da)	28200	33291	31274	33292
M from program SAXS (Da)	26500	36700	30800	36000
**Structural parameters**				
$I(0)$ (cm^{-1})	0.12	0.11	0.10	0.11
R_g (Å) (Guinier)	21.94	26.90	22.19	26.63
q_{min} (Å^{-1})	0.016	0.016	0.030	0.020
qR_g max	1.30	1.30	1.29	1.30
Coefficient of correlation, R^2	0.990	0.993	0.997	0.998
d_{max} (Å)	56	65	59	62
**Software employed**				
Data processing Computation of model intensities	FoXS [5]	FoXS	FoXS	FoXS
SAXS data reduction	RAW [6]	RAW	RAW	RAW
Three-dimensional graphics representations	PyMOL [7]	PyMOL	PyMOL	PyMOL

Table | S4. PISA analysis of the interaction between CTD and the other part of NMAD-1A

Structure 1	Structure 2	Interface	ΔG^{diss}
NMAD-1A$_{21\text{-}263}$	NMAD-1A$_{264\text{-}291}$ (CTD)	959.7 Å^2	9.5 kcal/mol

ΔG^{diss} indicates the free energy of assembly dissociation, in kcal/mol.

References:

1. Zhang, M., Yang, S., Nelakanti, R., Zhao, W., Liu, G., Li, Z., Liu, X., Wu, T., Xiao, A., and Li, H. Mammalian ALKBH1 serves as an N(6)-mA demethylase of unpairing DNA. *Cell Res.* **2020**, *30*, 197-210.
2. Notredame, C., Higgins, D. G., and Heringa, J. T-Coffee: A novel method for fast and accurate multiple sequence alignment. *J. Mol. Biol.* **2000**, *302*, 205-217.
3. Magis, C., Taly, J. F., Bussotti, G., Chang, J. M., Di Tommaso, P., Erb, I., Espinosa-Carrasco, J., and Notredame, C. T-Coffee: Tree-based consistency objective function for alignment evaluation. *Methods Mol. Biol.* **2014**, *1079*, 117-129.
4. Zacharias, J., and Knapp, E. W. Protein secondary structure classification revisited: processing DSSP information with PSSC. *J. Chem. Inf. Model.* **2014**, *54*, 2166-2179.
5. Schneidman-Duhovny, D., Hammel, M., and Sali, A. FoXS: a web server for rapid computation and fitting of SAXS profiles. *Nucleic Acids Res.* **2010**, *38*, W540-544.
6. Hopkins, J. B., Gillilan, R. E., and Skou, S. BioXTAS RAW: improvements to a free open-source program for small-angle X-ray scattering data reduction and analysis. *J. Appl. Crystallogr.* **2017**, *50*, 1545-1553.
7. Alexander, N., Woetzel, N., and Meiler, J. (2011) Bcl::Cluster: A method for clustering biological molecules coupled with visualization in the Pymol Molecular Graphics System. in *IEEE Int. Conf. Comput. Adv. Bio. Med. Sci.* **2011**, *2011*, 13-18.
